# Hypoxia-Inducible Factor Directs POMC Gene to Mediate Hypothalamic Glucose Sensing and Energy Balance Regulation

**DOI:** 10.1371/journal.pbio.1001112

**Published:** 2011-07-26

**Authors:** Hai Zhang, Guo Zhang, Frank J. Gonzalez, Sung-min Park, Dongsheng Cai

**Affiliations:** 1 Department of Molecular Pharmacology, Albert Einstein College of Medicine, Bronx, New York, United States of America; 2 Department of Physiology, University of Wisconsin–Madison, Madison, Wisconsin, United States of America; 3 Cellular & Molecular Biology Program, University of Wisconsin–Madison, Madison, Wisconsin, United States of America; 4 Center for Cancer Research, National Cancer Institute, Bethesda, Maryland, United States of America; 5 Dominick P. Purpura Department of Neuroscience, Albert Einstein College of Medicine, Bronx, New York, United States of America; 6 Diabetes Research Center, Albert Einstein College of Medicine, Bronx, New York, United States of America; University of Cambridge, United Kingdom

## Abstract

Hypoxia-inducible factor (HIF) is a nuclear transcription factor that responds to environmental and pathological hypoxia to induce metabolic adaptation, vascular growth, and cell survival. Here we found that HIF subunits and HIF2α in particular were normally expressed in the mediobasal hypothalamus of mice. Hypothalamic HIF was up-regulated by glucose to mediate the feeding control of hypothalamic glucose sensing. Two underlying molecular pathways were identified, including suppression of PHDs by glucose metabolites to prevent HIF2α degradation and the recruitment of AMPK and mTOR/S6K to regulate HIF2α protein synthesis. HIF activation was found to directly control the transcription of *POMC* gene. Genetic approach was then employed to develop conditional knockout mice with HIF inhibition in POMC neurons, revealing that HIF loss-of-function in POMC neurons impaired hypothalamic glucose sensing and caused energy imbalance to promote obesity development. The metabolic effects of HIF in hypothalamic POMC neurons were independent of leptin signaling or pituitary ACTH pathway. Hypothalamic gene delivery of HIF counteracted overeating and obesity under conditions of nutritional excess. In conclusion, HIF controls hypothalamic *POMC* gene to direct the central nutrient sensing in regulation of energy and body weight balance.

## Author Summary

The hypothalamus in the brain is a master regulator of feeding and body weight. The regulation of it is mediated by the ability of the hypothalamus to sense nutrients (most importantly glucose) and hormones (such as insulin and leptin). While hormone has been extensively studied, we know less about how the hypothalamus can sense nutrients. It is also unclear whether changes in hypothalamic nutrient sensing can influence the development of obesity and related disease, and could therefore be targeted for disease intervention. In this study, we show that a protein termed hypoxia-inducible factor (HIF) is normally present in the hypothalamus and able to respond to glucose. This glucose response leads to the up-regulation of a hypothalamic neuropeptide, POMC, a pivotal molecule that controls feeding and body weight balance. We then developed a mouse model in which HIF is disrupted in hypothalamic cells that express POMC. These mice displayed reduced hypothalamic sensitivity to glucose, resulting in overeating and susceptibility to obesity. Furthermore, we found that delivery of the HIF gene into the hypothalamus has strong anti-obesity effects in mice. We conclude that HIF is a molecular mediator of hypothalamic glucose sensing and can be potentially targeted for obesity therapeutics.

## Introduction

Hypoxia-inducible factor (HIF) is the central nuclear transcription factor that is induced under environmental (e.g., high altitude) and pathological (e.g., cancer) hypoxia [1],[2]. HIF exists as the heterodimer of an α subunit and a β subunit; HIFα protein levels are regulated based on tissue oxygen availability, while HIFβ (also called aryl hydrocarbon receptor nuclear translocator, or ARNT) is constitutively present [1]–[4]. Under normoxia, HIFα undergoes protein hydroxylation, ubiquitination, and degradation, and this process is mediated by prolyl hydroxylases (PHDs) and ubiqutin E3 ligase pVHL—the product of von Hippel-Lindau (*VHL*) gene [1]–[4]. Under hypoxia, PHDs are suppressed, leading to HIFα protein stabilization and thus the transcriptional action of HIFα/β in inducing genes that classically regulate metabolic adaptation, vascular growth, and cell survival [1]–[4]. Among three HIFα isoforms (HIF1α, HIF2α, and HIF3α), HIF1α and HIF2α have been extensively studied in the literature [1]–[4]. While both HIF1α and HIF2α mediate hypoxia adaptation, HIF2α can control a distinct set of target genes [5],[6]. Consistently, the biological consequences of HIF2α versus HIF1α ablation in mice are different [7]–[9], suggesting that HIF2α and HIF1α have divergent physiological functions.

Recent research has elucidated that regulation of HIF1/2α by hypoxia involves metabolic mediators, such as reactive oxygen species that can modulate mitochondrial complex III [1]–[4],[10]–[12]. In addition, HIF is subjected to normoxic regulation, and the underlying basis has been related to several metabolic signaling pathways including the PI3K-mTOR cascade [13]–[15] and SIRT1 [16]. The biochemical regulation of HIF by various metabolic signals has been implicated to underlie the metabolic programming of tumorigenesis [1]–[4]. However, it remains unexplored whether a reverse relationship might exist, i.e., if HIF could be a primary regulator of metabolic physiology at an organism level and hence a critical target for controlling metabolic disease, and if so, what could be the responsible tissue/cell types and the underlying molecular basis.

The hypothalamus in the central nervous system (CNS) is the master regulator of energy intake (feeding), energy expenditure, and body weight balance [17]–[21]. The responsible neuronal regulation involves not only hormonal sensing by molecules such as leptin and insulin [17]–[21] but also nutrient sensing by species such as glucose, amino acid, and fatty acids [22]–[32]. Compared to the long-term homeostatic regulation of body weight by hypothalamic hormonal signaling, hypothalamic glucose sensing is rapid and predicted to provide an acute and real-time regulation on metabolic homeostasis. Proopiomelanocortin (POMC)-expressing neurons, termed POMC neurons, have been identified to account for hypothalamic glucose sensing [27],[33]. In this report, we demonstrate that hypothalamic glucose sensing is mediated by HIF activation and resulting up-regulation of *POMC* gene and that HIF loss-of-function in POMC neurons causes glucose desensitization to promote energy imbalance and obesity development.

## Results

### HIF Controls POMC Gene Transcription

**Figure 1:**
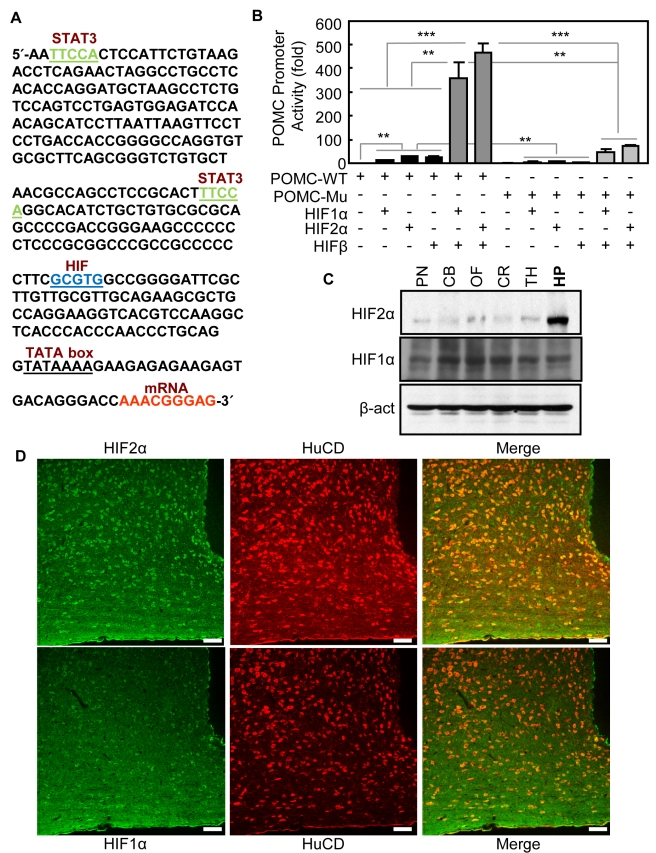
Regulation of HIF on POMC gene and HIF profile in the hypothalamus. (A) Analysis of the proximal region of rat POMC promoter. Putative HIF binding element, STAT3 binding element, TATA box, and initiation site of mRNA transcription are indicated. (B) HEK 293 cells were transfected with firefly luciferase pGL3 controlled by either wildtype (WT) or mutant (Mu) rat POMC promoter, and control renilla luciferase pRL-TK, together with the indicated combination of pcDNA-HIF1α, pcDNA-HIF2α, pcDNA-HIFβ, and empty pcDNA. Mutant POMC promoter was generated by deleting 5′-GCGTG-3′ in the WT version of *POMC* promoter. ** *p*<0.01, *** *p*<0.001; *n* = 4 per group. Error bars reflect mean ± SEM. (C) HIF2α and HIF1α expression levels in different brain areas of C57BL/6 mice were analyzed using Western blotting. β-actin (β-act) was used as a control. PN, pons; CB, cerebellum; OF, olfactory bulb; CR, cortex; TH, thalamus; HP, hypothalamus. (D) Brain sections of the mediobasal hypothalamus were prepared from regular C57BL/6 mice and immunostained with HIF2α antibody (upper panel) or HIF1α antibody (lower panel). HIF2α or HIF1α (green) was co-immunostained with neuronal marker HuCD (red). Merged images (yellow/orange) revealed neuronal expression of HIF2α or HIF1α protein. 3V, third ventricle; ARC, arcuate nucleus. Bar = 50 µm.

In order to screen nuclear transcription factors that control hypothalamic neuropeptide genes, we analyzed the DNA sequence of *POMC* promoter and identified a HIF-responsive element at the proximal promoter region across species from rodents to humans. POMC is the precursor of hypothalamic neuropeptide, α-melanocyte-stimulating hormone (α-MSH), which is an important hypothalamic regulator of feeding and energy balance, and mutation of *POMC* gene is sufficient to cause severe obesity and diabetes in both rodents [34] and humans [35],[36]. Notably, the HIF-binding DNA element (5′-GCGTG-3′) is located immediately upstream of the transcriptional initiation site (TATA box) in the *POMC* promoter (Figure 1A). In contrast, the DNA elements for STAT3, the most established nuclear transcription factor for *POMC* gene in leptin signaling [37]–[39], are located further upstream. Using a luciferase reporter system, we found that overexpression of HIF1α and HIF2α increased the activities of transfected *POMC* promoter by 12 folds and 26 folds, respectively (Figure 1B). When HIF1α or HIF2α was co-overexpressed with HIFβ, the heterodimeric complex activated *POMC* promoter by 362–466-fold (Figure 1B). On the other hand, deletion of the 5-bp HIF-binding DNA element (5′-GCGTG-3′) substantially prevented HIF from activating the mutant *POMC* promoter (Figure 1B). All these data suggest that POMC gene represents a HIF target.

### Differential Patterns of HIF1α and HIF2α in the Hypothalamus

To explore whether there was an anatomic basis to support a metabolic role of hypothalamic HIF, we then profiled HIF isoform distribution in the hypothalamus as well as other brain regions. Western blot analysis of HIF2α showed high protein levels in the hypothalamus but low levels in many other brain regions, including cortex, thalamus, olfactory bulb, pons, and cerebellum (Figure 1C). Unlike HIF2α, HIF1α protein expression was normally weak throughout the brain (Figure 1C). We then performed brain immunostaining of HIF2α versus HIF1α. The specificity of HIF2α and HIF1α antibodies for immunostaining were both verified through exogenous expression and co-immunostaining with conjugated epitope tags. In these experiments, hypothalamic GT1-7 cells were transfected with pcDNA3.1 plasmid, which expressed myc-tagged HIF2α or myc-tagged HIF1α. The induction of HIFα isoforms was detected by the immunostaining of anti-HIF1α or anti-HIF2α antibody, and the specificity was confirmed by co-immunostaining with anti-myc antibody. The results showed that anti-HIF2α antibody (Figure S1) and anti-HIF1α antibody (Figure S2) provided equal sensitivity and did not yield cross-reactions. Subsequently, we employed these two antibodies to map HIF2α versus HIF1α in the brain of normal C57BL/6 mice. Immunostaining revealed that HIF2α was abundant in neurons of the mediobasal hypothalamus that comprised the arcuate nucleus (Figure 1D), but less abundant in many other brain regions (unpublished data). Compared to HIF2α, HIF1α was weakly expressed in the hypothalamus (Figure 1D) and barely detectable in many other brain regions. Altogether, these data suggest that HIFα, in particular HIF2α, might be involved in hypothalamic regulation of whole-body physiology.

### HIF Inhibition in POMC Neurons Abrogates Feeding Regulation of Glucose Sensing

**Figure 2:**
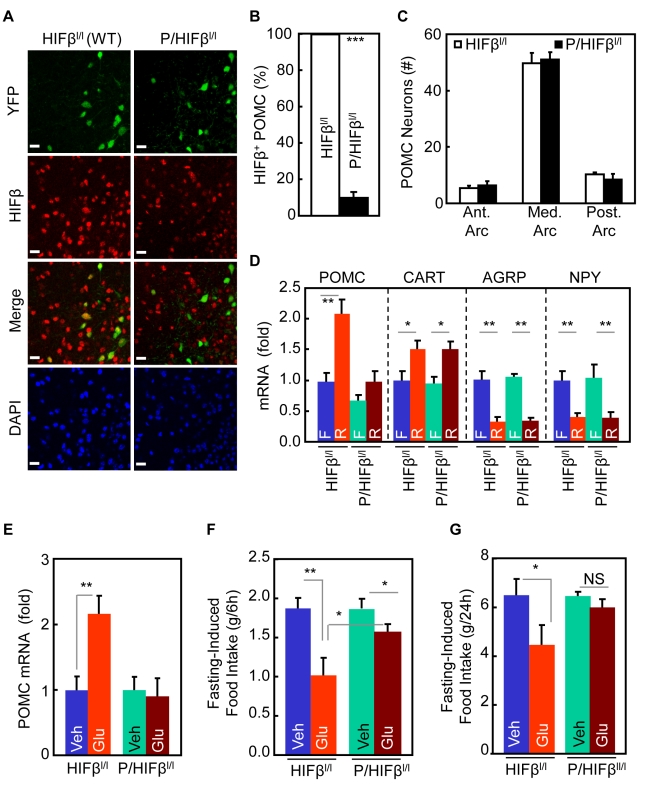
HIF inactivation in POMC neurons impairs glucose regulation of feeding. (A&B) Hypothalamic sections from *POMC/HIFβ^lox/lox^* mice and control *HIFβ^lox/lox^* mice were stained with HIFβ (red). YFP (green) was introduced to these mice (via crossing these mice with *ROSA-flox-STOP-flox-YFP* mice) to visualize POMC neurons. DAPI staining shows nuclei of all cells in the sections. Bar = 25 µm. Graphs: percentage of HIFβ-positive POMC neurons. *** *p*<0.001; *n* = 3–4 per group. Error bars reflect mean ± SEM. (C) Counting analysis for the total numbers of POMC neurons across anterior, medial, and posterior arcuate nucleus of *POMC/HIFβ^lox/lox^* mice versus control *HIFβ^lox/lox^* mice. *n* = 4 per group. Error bars reflect mean ± SEM. (D) 24-h fasted young *POMC/HIFβ^lox/lox^* mice (*P/HIFβ^l/l^*) and control littermate *HIFβ^lox/lox^* mice (*HIFβ^l/l^*) received 6-h re-feeding (R) versus continued 6-h fasting (F). Hypothalami were subsequently harvested for the measurement of neuropeptide mRNA levels. (E) 24-h fasted young *POMC/HIFβ^lox/lox^* mice (*P/HIFβ^l/l^*) and control littermate *HIFβ^lox/lox^* mice (*HIFβ^l/l^*) received injection of glucose (Glu) versus vehicle (Veh) via cannula pre-implanted into the third ventricle. Hypothalami were then harvested for the measurement of POMC mRNA. (F&G) Young, male *POMC/HIFβ^lox/lox^* mice (*P/HIFβ^lox/lox^* mice) versus littermate control *HIFβ^lox/lox^* mice were fasted 24 h and received third-ventricle injection of glucose or vehicle. Food was placed in cages, and mice were subsequently monitored for food intake for 6 h (F) and 24 h (G). (D–G) * *p*<0.05, ** *p*<0.01, NS, non-significant; *n* = 6–14 per group. Error bars reflect mean ± SEM.

POMC neurons have been known as a major hypothalamic neuronal type that mediates glucose sensing of the hypothalamus [27],[33]. Hence, we investigated whether HIF inactivation in POMC neurons could affect hypothalamic glucose sensing in regulation of feeding. To test this question, we chose to ablate *HIFβ* gene, since HIFβ is mandatory for the DNA binding and activation of both HIF1 and HIF2 complexes [1]–[4]. By crossing *POMC-Cre* mice [40] with *HIFβ^lox/lox^* mice [41], we created a knockout mouse model with *HIFβ* gene ablated in hypothalamic POMC neurons, termed *POMC/HIFβ^lox/lox^* mice. To evaluate the efficiency of *HIFβ* ablation in the knockout mice, we further crossed *POMC/HIFβ^lox/lox^* mice with *ROSA-flox-STOP-flox-YFP* mice in order to visualize POMC neurons in brain sections. Using this tool, we revealed that HIFβ protein was disrupted in the majority (∼90%) of POMC neurons in *POMC/HIFβ^lox/lox^* mice (Figure 2A&B). The specificity of anti-HIFβ antibody was verified in cultured cells by co-staining of transfected HIFβ with the conjugated tag (Figure S3). The total number and morphology of hypothalamic POMC neurons were not affected by *HIFβ* gene ablation (Figure 2C), suggesting that HIF inactivation did not impair the development of POMC neurons.

*POMC/HIFβ^lox/lox^* mice were developmentally indistinguishable from littermate control *HIFβ^lox/lox^* mice. *POMC/HIFβ^lox/lox^* mice and controls at young ages (1∼3 mo old) had similar body weight. This knockout mouse model was then employed to test whether *HIFβ* ablation could compromise nutrient-induced hypothalamic *POMC* mRNA expression. Two experimental paradigms were used: supply of general nutrients through re-feeding post-fasting and glucose administration through third-ventricle infusion. First, it was found that re-feeding post-fasting significantly increased hypothalamic levels of *POMC* mRNA in control mice, but failed to do so in *POMC/HIFβ^lox/lox^* mice (Figure 2D). *HIFβ* ablation did not alter mRNA levels of hypothalamic neuropeptides *CART*, *AGRP*, and *NPY* (Figure 2D) or hindbrain neuropeptide *nesfatin-1* (Figure S4A). We also examined a few other nuclear transcription factors including *BSX*, *FoxO1*, and *CREB*, which can also control neuropeptide expression. The expression levels of these genes were unchanged in the hypothalamus of *POMC/HIFβ^lox/lox^* mice compared to controls (Figure S4B). Second, we performed the experiment using third-ventricle glucose infusion. Similar to re-feeding, glucose infusion up-regulated hypothalamic *POMC* mRNA levels in control mice; however, this up-regulation was not induced in *POMC/HIFβ^lox/lox^* mice (Figure 2E). In sum, the data suggested that HIF mediates glucose-dependent hypothalamic POMC gene expression.

Subsequently, we investigated whether the *HIFβ* ablation could compromise the feeding-restricting effect of hypothalamic glucose sensing. Following a prolonged fasting (24 h), *POMC/HIFβ^lox/lox^* mice and their littermate controls received third-ventricle injection of glucose via pre-implanted cannula. Indeed, glucose suppressed fasting-induced feeding in control mice. This anorexic effect occurred rapidly within 6 h post-injection (Figure 2F) and lasted throughout 24-h follow-up period (Figure 2G). In contrast, glucose-induced appetite suppression was substantially abolished in *POMC/HIFβ^lox/lox^* mice (Figure 2F&G). Hence, HIF in POMC neurons is required for glucose-dependent hypothalamic regulation of feeding behavior.

### Absence of Pituitary Changes in POMC/HIFβ^lox/lox^ Mice

POMC cells are present in the hypothalamus as well as the pituitary; thus, both places were targeted by the Cre-loxp technique for *HIFβ* ablation in the knockout mice. Following the above observations in *POMC/HIFβ^lox/lox^* mice, we examined whether the pituitary POMC cells were affected by *HIFβ* ablation in the knockout mice. Because pituitary POMC is the precursor of adrenocorticotropic hormone (ACTH), we evaluated the pituitary ACTH synthesis via ACTH immunostaining. Data revealed that the numbers of pituitary ACTH-positive cells and ACTH expression levels were similar between *POMC/HIFβ^lox/lox^* mice and littermate controls (Figure S5A&B). Consistently, pituitary morphology (Figure S5A), ACTH release (Figure S5C), and pituitary mass (Figure S5D) in the *POMC/HIFβ^lox/lox^* mice were normal. Also, since the main function of ACTH is to control adrenal growth and corticosterone release, we analyzed the histology of adrenal glands from *POMC/HIFβ^lox/lox^* mice and matched controls. Indeed, the adrenal morphology and mass were comparable between *POMC/HIFβ^lox/lox^* mice and the controls (Figure S5E&F). In line with this profile, blood corticosterone concentrations in *POMC/HIFβ^lox/lox^* mice and controls were also similar (Figure S5G). Altogether, these data indicated that the pituitary POMC-ACTH system was not involved in glucose-related feeding dysregulation of *POMC/HIFβ^lox/lox^* mice.

### Glucose Suppresses PHDs to Stabilize Hypothalamic HIF2α

**Figure 3 pbio-1001112-g003:**
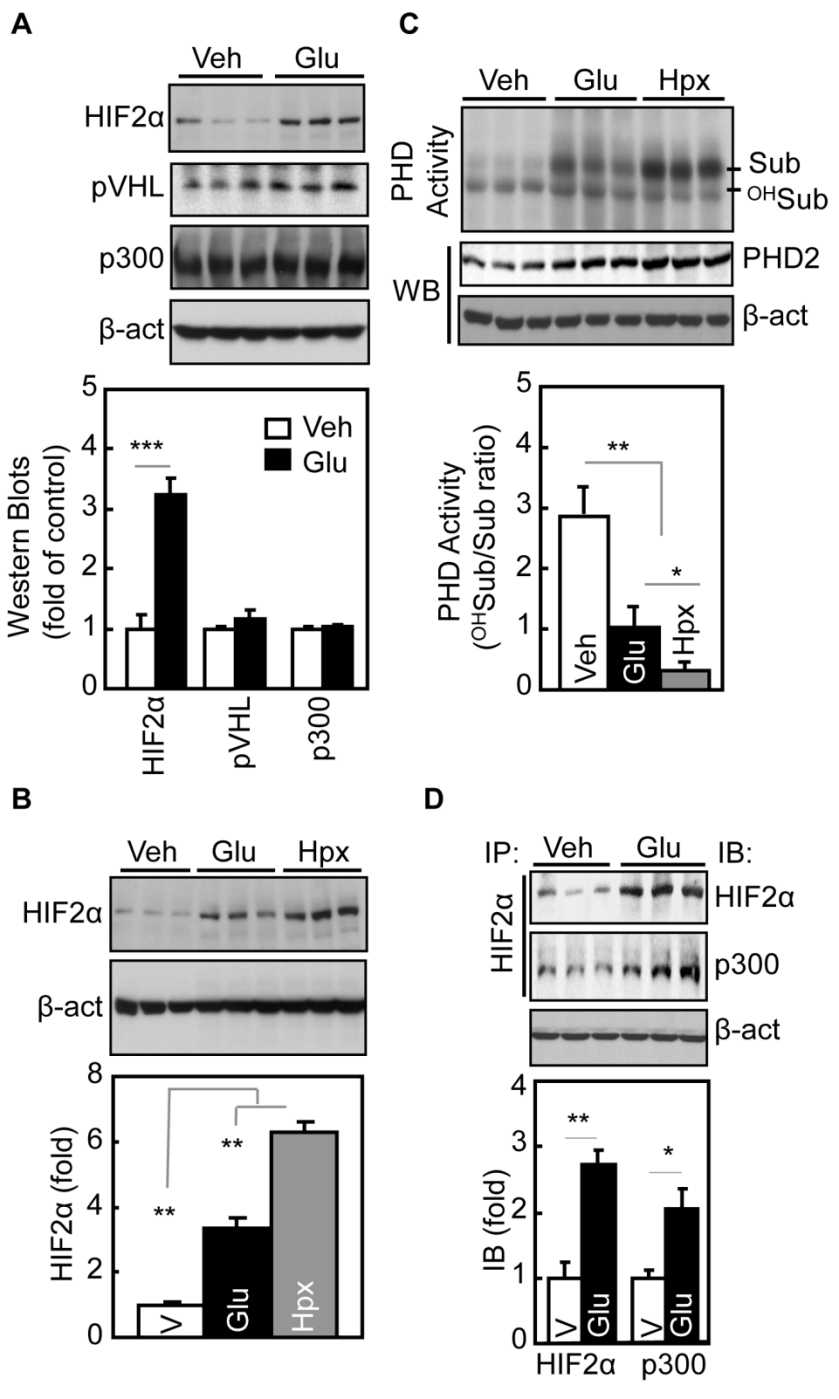
Regulation of hypothalamic HIF by glucose. (A) 24-h fasted adult C57BL/6 mice received 5-h third-ventricle infusion of glucose (Glu) or vehicle (Veh), and hypothalami were analyzed by Western blots. Bar graphs: Western blot results were normalized by β-actin (β-act) and analyzed statistically. *** *p*<0.001, *n* = 6 per group. (B) 24-h fasted regular C57BL/6 mice received third-ventricle infusion of glucose (Glu) versus vehicle (Veh). Positive controls included 6-h hypoxia (Hpx) treatment. Hypothalami were then harvested for Western blot analysis of HIF2α protein levels. β-actin (β-act) was used as an internal control. Bar graphs: HIF2α protein levels were normalized by β-actin and analyzed statistically. ** *p*<0.01; *n* = 5–6 per group. Error bars reflect mean ± SEM. (C) 24-h fasted adult C57BL/6 mice received 5-h third-ventricle infusion of glucose (Glu) versus vehicle (Veh). Positive controls included matched mice that received 6-h hypoxia (Hpx) treatment. Hypothalami were dissected and lysed for the measurement of PHD activity using the GHO assay. PHD activity is expressed by the ratio of hydroxylated GHO substrate (^OH^Sub) to intact GHO substrate (Sub). PHD2 and β-actin levels in the lysates were also measured using Western blots. Bar graphs: quantitative analysis of PHD activities. * *p*<0.05, ** *p*<0.01; *n* = 6 per group. Error bars reflect mean ± SEM. (D) 24-h fasted regular C57BL/6 mice received third-ventricle infusion of glucose (Glu) versus vehicle (Veh). Hypothalami were isolated, lysed, and analyzed for co-immunoprecipitation of endogenous HIF2α and p300 using HIF2α immunoprecipitation (IP) followed by HIF2α and p300 immunoblotting (IB) detection. Protein lysates were examined by Western blots for β-actin (β-act). Bar graphs: immunoprecipitated HIF2α and p300 were normalized by β-actin and analyzed statistically. * *p*<0.05, ** *p*<0.01; *n* = 5–6 per group. Error bars reflect mean ± SEM. (A–D) All mice were male C57BL/6 under normal chow feeding.

The next question was: How could glucose activate hypothalamic HIF? Since mRNA levels of hypothalamic *HIF2α* and *HIF1α* were not affected by third-ventricle glucose infusion (Figure S4C), glucose regulation of hypothalamic HIF was not mediated via *HIF* mRNA expression. In contrast, third-ventricle glucose infusion significantly increased HIF2α protein levels in the hypothalamus (Figure 3A&B, Figure S6A). This effect was not evident in peripheral tissues of mice that were i.p. injected with glucose (Figure S6B&C). To understand the molecular basis for glucose-induced hypothalamic HIF up-regulation, we tested if it involved PHDs-dependent HIFα hydroxylation and degradation—which is the classical molecular cascade in regulation of HIF activity [1],[2]. Using the GHO assay, which was established in the literature [42], we found that third-ventricle glucose delivery suppressed the hydroxylation activities of PHDs in the hypothalamus (Figure 3C). We also examined pVHL, an E3 ubiquitin ligase that mediates ubiquitination and degradation of hydroxylated HIFα. Glucose did not change pVHL protein levels in the hypothalamus (Figure 3A), suggesting that pVHL was not a primary factor for glucose-induced HIFα up-regulation. Thus, glucose employs the PHD-pVHL system to induce hypothalamic HIF up-regulation, although the magnitude of this effect was much smaller than that of hypoxia (Figure 3B&C). We also analyzed the binding of HIF2α to p300, since the complex functions in the nucleus to exert the transcriptional activity. As shown in Figure 3D, increased hypothalamic HIF2α protein levels were proportionally associated with the increased binding of HIF2α to p300, suggesting that up-regulation of hypothalamic HIF2α by glucose is transcriptionally functional.

### HIF Mediates Hypothalamic Glucose Sensing Without Involving Leptin Signaling

We further asked whether HIF2α might be involved in the action of leptin, a well-established hormone that employs nuclear transcription factor STAT3 to mediate hypothalamic regulation of feeding [17]–[19]. First, in contrast with the effect of glucose, leptin administration via the third ventricle did not alter HIF2α protein levels in the hypothalamus (Figure S7A). Then, we investigated whether HIF might be required for the signaling and function of leptin in the hypothalamus. To test this question, we employed a loss-of-function strategy by analyzing hypothalamic leptin signaling and leptin-dependent feeding regulation in *POMC/HIFβ^lox/lox^* mice. Compared to the control mice, *POMC/HIFβ^lox/lox^* mice showed similar levels of leptin-induced STAT3 phosphorylation in the hypothalamus including the comprised POMC neurons (Figure S7C&D). Thus, hypothalamic HIF was responsive to glucose but not leptin, which aligns with the observation that hypothalamic glucose sensing did not involve the induction of leptin signaling (Figure S7B). To evaluate the physiological relevance of this finding, experiments were performed to compare leptin-dependent feeding regulation in *POMC/HIFβ^lox/lox^* mice versus control mice. Data showed that food intake in *POMC/HIFβ^lox/lox^* mice and the matched controls were suppressed by leptin in a similar manner (Figure S7E). Altogether, while STAT3 is a critical nuclear transcription factor in leptin signaling, HIF represents a nuclear transcription factor that crucially mediates the glucose-sensing process of the hypothalamus.

### Glucose Up-Regulates Hypothalamic HIF2α via Fumarate and Succinate

**Figure 4 pbio-1001112-g004:**
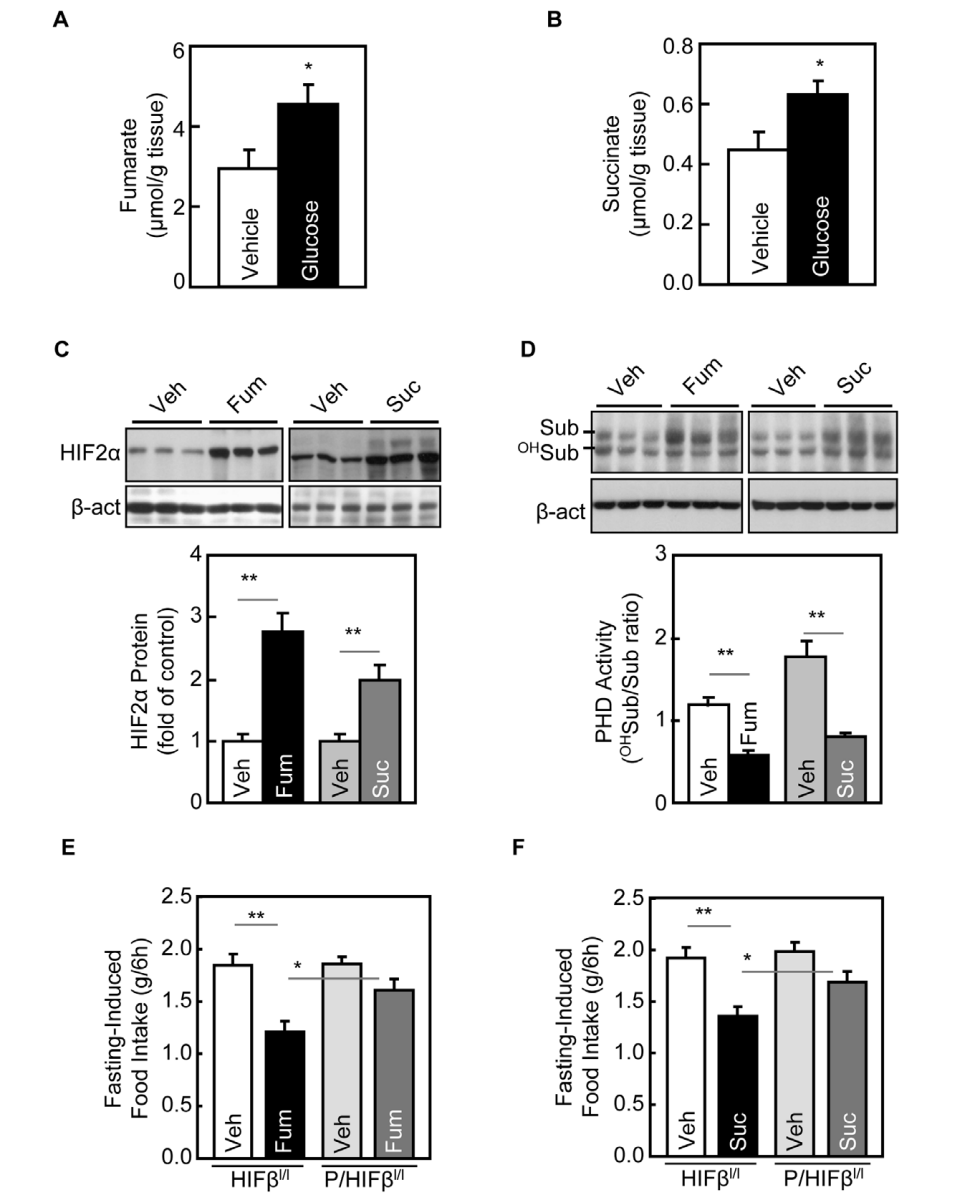
Glucose metabolites mediate glucose regulation of hypothalamic HIF. (A&B) 24-h fasted C57BL/6 mice received third-ventricle infusion of glucose versus vehicle. Hypothalami were harvested and measured for tissue contents of fumarate (A) and succinate (B). * *p*<0.05; *n* = 5–6 per group. Error bars reflect mean ± SEM. (C&D) 24-h fasted C57BL/6 mice received third-ventricle infusion of diethyl fumarate (Fum) or diethyl succinate (Suc) or control vehicle (Veh). Hypothalami were harvested for Western blot analysis of HIF2α protein (C) and GHO assay for PHD activities (D). Bar graphs: HIF2α protein levels (C) and PHD activities (D) were quantitated and analyzed statistically. ** *p*<0.01; *n* = 5–6 per group. Error bars reflect mean ± SEM. (E&F) 24-h fasted *POMC/HIFβ^lox/lox^* mice (*P/HIFβ^l/l^*) and control littermates *HIFβ^lox/lox^* mice (*HIFβ^l/l^*) received third-ventricle injection of diethyl fumarate (Fum) (E), diethyl succinate (Suc) (F), or vehicle (Veh) (E&F), and subsequently monitored for food intake for 6 h. * *p*<0.05; ** *p*<0.01; *n* = 7–10 per group. Error bars reflect mean ± SEM. (A–F) All mice were adult males and normal chow-fed.

It has been recently demonstrated that glucose metabolites fumarate and succinate can inhibit PHDs to activate HIF in cancer cells to promote tumorigenesis [4]. Hinted by this information, we questioned if fumarate and succinate could mediate glucose-dependent HIF activation in the hypothalamus. To examine this idea, we first confirmed the prediction that glucose delivery via the third ventricle increased the production of fumarate and succinate in the hypothalamus (Figure 4A&B). Then, after having established the appropriate dose- and time-course conditions (Figure S8A–D), we revealed that HIF2α protein levels in the hypothalamus of normal C57LB/6 mice were significantly increased by a third-ventricle delivery of either fumarate or succinate (Figure 4C, Figure S8E&F). Furthermore, fumarate and succinate were both found to suppress the PHD hydroxylation activities in the hypothalamus (Figure 4D). These data indicated that these two glucose metabolites activated hypothalamic HIF via the PHD-pVHL pathway.

Subsequently, we examined whether manipulation of hypothalamic fumarate or succinate could affect feeding in mice. As established in the literature [43],[44], succinate and fumarate can be accumulated by using inhibitors of either a succinate dehydrogenase, thenoyltrifluoroacetone (TTFA), or a fumarate hydratase inhibitor, trans-aconitate, or 3-nitropropionic acid (3-NPA). We found that individual delivery of these chemicals via third-ventricle injection inhibited fasting-induced food intake of mice (Figure S9A) without evident toxic/aversive effects (Figure S9B). Then, we tested if succinate or furmarate administration into the third ventricle of mice could affect their feeding activities. Indeed, we found that a third-ventricle injection of either fumarate or succinate suppressed food intake in the control mice. In contrast, such effects were significantly reduced in *POMC/HIFβ^lox/lox^* mice (Figure 4E&F). Hence, fumarate and succinate are two glucose metabolites that can mediate glucose up-regulation of hypothalamic HIF.

### AMPK Down-Regulation by Glucose Mediates Hypothalamic HIF Activation

**Figure 5 pbio-1001112-g005:**
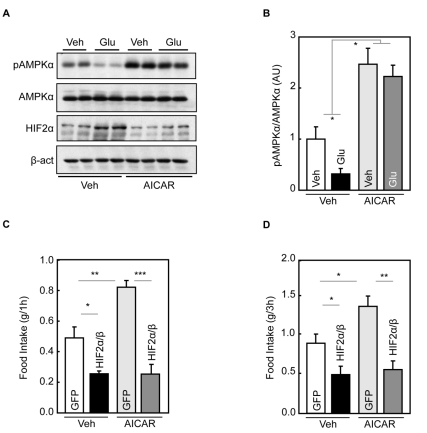
Effect of AMPK in glucose-mediated activation of hypothalamic HIF. (A&B) Western blot analyses of AMPK signaling were performed for the hypothalami harvested from C57BL/6 mice that received a 5-h intra-third ventricle infusion of glucose (Glu) versus vehicle (Veh) in the presence or absence of a prior third-ventricle injection of AICAR. β-act, β-actin. Bar graphs: Western blots were quantitated and analyzed statistically. (C&D) C57BL/6 mice received mediobasal hypothalamic injection of neuron-specific lentiviruses expressing HIF2α/HIFβ (HIF2α/β) or GFP, and simultaneously received cannula implantation into the third ventricles. After 2-wk post-surgical recovery, mice received 6-h fasting and then were injected with AICAR or vehicle (Veh) via the cannula. Mice subsequently had free access to food and were measured for food intake during 1-h (C) and 3-h (D) periods. (B–D) * *p*<0.05, ** *p*<0.01, *** *p*<0.001; *n* = 6 per group. Error bars reflect mean ± SEM.

Recent research has revealed AMPK as an “energy gauge” in hypothalamic regulation of energy balance [30]–[32]. In this context, we asked if AMPK could be involved in hypothalamic HIF signaling and action. Using AMPKα phosphorylation to reflect AMPK activity, we found that an intra-third ventricle injection of glucose inhibited hypothalamic AMPK (Figure 5A&B). Then, we performed intra-third-ventricle injection of glucose in the presence or absence of AICAR, an established AMPK activator. Data revealed that AICAR markedly reduced the effect of glucose in hypothalamic HIF up-regulation (Figure 5A&B). This result indicated AMPK might work as an inhibitory regulator for glucose-dependent HIF activation in the hypothalamus.

Also, it has recently been reported that activation of hypothalamic AMPK by AICAR can promote food intake in mice [45],[46]. After having confirmed this effect in our experiment, we tested whether hypothalamic HIF could prevent the feeding-promoting effects of AICAR. To do this, we delivered HIF2α/HIFβ complex into the neurons in the mediobasal hypothalamus, since POMC neurons are predominantly localized in this region. Through a neuron-specific lentiviral vector in which dual synapsin promoters were used to direct co-expression of two genes (Figure S10A), HIF2α and HIFβ were co-delivered into the mediobasal hypothalamus of standard C57BL/6 mice (Figure S10B&C). Mice receiving lentiviral delivery of GFP were used as controls (Figure S10B). As shown in Figure 5C&D, a third-ventricle administration of AICAR promoted food intake in control mice as expected; however, this effect of AICAR was eliminated by the exogenous expression of HIF2α/HIFβ complex. Thus, AMPK suppression by glucose is mechanistically involved in glucose sensing of the hypothalamic HIF pathway.

### Up-regulation of mTOR/S6K by Glucose Mediates Hypothalamic HIF Activation

**Figure 6:**
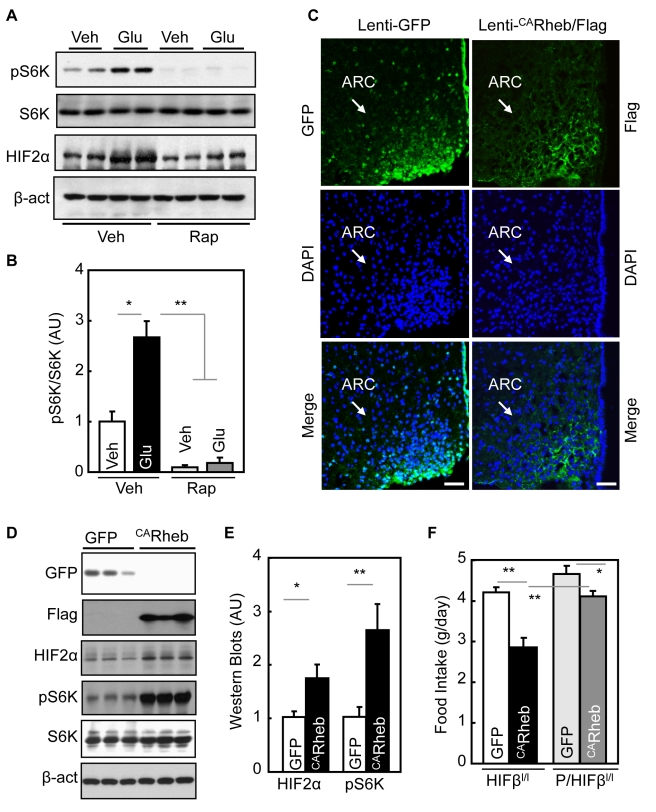
Regulation of mTOR pathway on hypothalamic HIF. (A&B) 24-h fasted adult C57BL/6 mice received 5-h third-ventricle infusion of glucose (Glu) versus vehicle (Veh) in the presence or absence a prior third-ventricle injection of rapamycin (Rap). Hypothalami were harvested and analyzed by Western blotting for phosphorylated S6K (pS6K) and HIF2α. β-act, β-actin. Bar graphs: Western blots were quantitated and analyzed statistically. * *p*<0.05, ** *p*<0.01; *n* = 4 per group. Error bars reflect mean ± SEM. AU, arbitrary unit. (C–F) *POMC/HIFβ^lox/lox^* mice (*P/HIFβ^l/l^*) and control littermate *HIFβ^lox/lox^* mice (*HIFβ^l/l^*) received MBH injection of lentiviruses expressing the constitutive-active form of Rheb tagged with Flag (Lenti-^CA^Rheb/Flag) or matched GFP-expressing control lentiviruses (Lenti-GFP). Site-specific gene delivery was verified by immunostaining (C) and Western blots of Rheb/mTOR signaling proteins (D) as well as the quantitation analysis of Western blots (E). Food intake of these mice was monitored on a daily basis for 2 wk (F). * *p*<0.05, ** *p*<0.01; *n* = 5–6 per group. Error bars reflect mean ± SEM. AU, arbitrary unit. (A–F) All mice were adult males and normal chow-fed.

We also examined the potential relevance of mTOR and its downstream component S6K, since mTOR/S6K can promote HIF1/2α protein synthesis in various experimental models [1],[2], and also AMPK can inhibit mTOR/S6K [47],[48]. We observed that glucose-dependent hypothalamic HIF2α up-regulation was associated with increased S6K activities (Figure 6A&B). Conversely, glucose induction of hypothalamic HIF2α was significantly reversed by third-ventricle injection of mTOR inhibitor, rapamycin (Figure 6A&B). Supported by recent research that has shown that hypothalamic mTOR [24],[49] and S6K [50] restrict feeding and weight gain, we further evaluated if mTOR/S6K might participate in the action of hypothalamic HIF in regulation of feeding. Using the site-specific gene delivery approach described above, we delivered lentiviruses expressing constitutively active Rheb (^CA^Rheb) to directly activate mTOR in the mediobasal hypothalamus of *POMC/HIFβ^lox/lox^* mice versus littermate controls (Figure 6C–F). As revealed in Figure 6F, while ^CA^Rheb decreased food intake in control mice, this effect was reduced in *POMC/HIFβ^lox/lox^* mice. In summary, glucose sensing of HIF in POMC neurons critically involves mTOR/S6K signaling.

### HIF Inhibition in POMC Neurons Causes Overfeeding and Energy Imbalance

**Figure 7:**
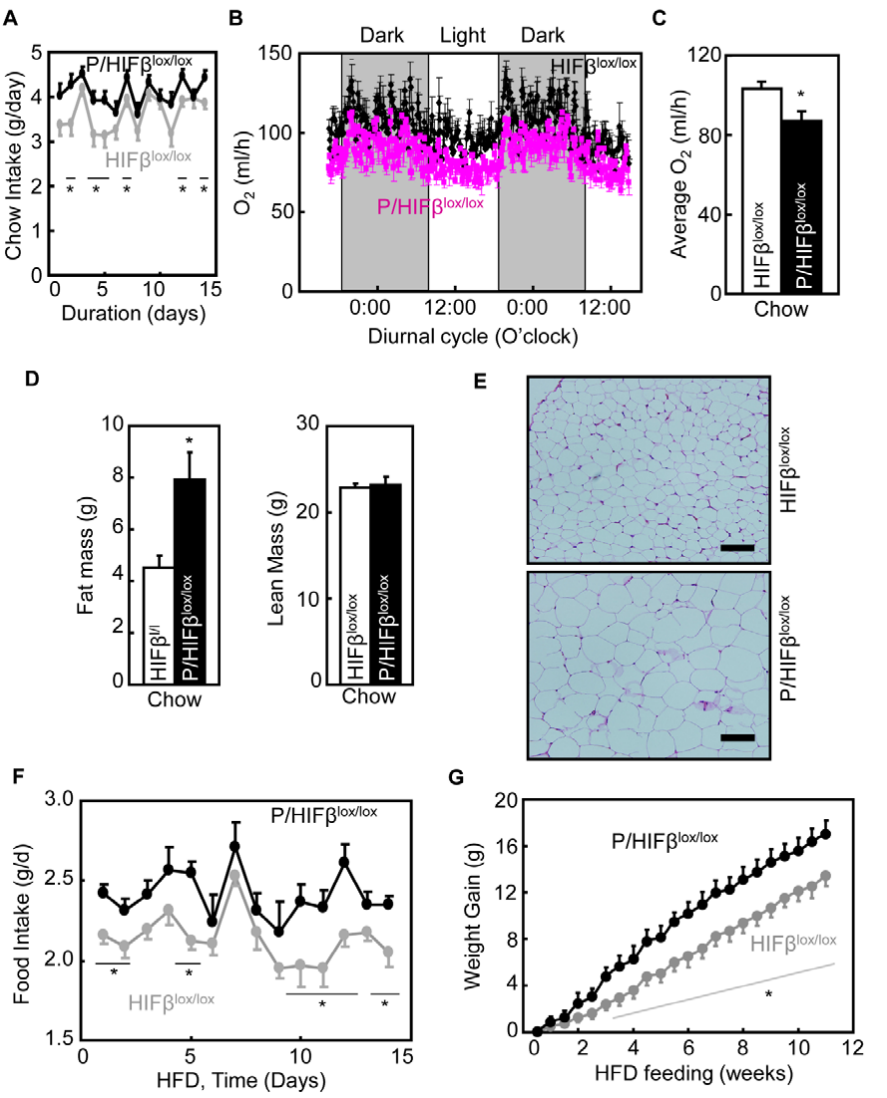
Inactivation of HIF in POMC neurons causes energy and metabolic imbalance. (A–C) Chow-fed *POMC/HIFβ^lox/lox^* mice (*P/HIFβ^lox/lox^*) and littermate control *HIFβ^lox/lox^* were analyzed for food intake (A) and O_2_ consumption (B&C). * *p*<0.05; *n* = 10 per group (A) and *n* = 4 per group (B&C). Statistics in (A) delineate the difference between two mice groups at the indicated time points. Error bars reflect mean ± SEM. (D) DEXA scanning of fat mass (versus lean mass) in chow-fed *POMC/HIFβ^lox/lox^* (*P/HIFβ^lox/lox^*) mice and littermate control *HIFβ^lox/lox^* (*HIFβ^l/l^*) mice. * *p*<0.05; *n* = 6 per group. (E) H&E staining of epididymal fat from normal chow-fed *POMC/HIFβ^lox/lox^* (*P/HIFβ^lox/lox^*) mice and littermate control *HIFβ^lox/lox^* mice. Bar = 100 µm. (F&G) HFD-fed *POMC/HIFβ^lox/lox^* mice (*POMC/HIFβ^lox/lox^*) and littermate control *HIFβ^lox/lox^* mice were monitored for food intake (F) and weight gain (G). * *p*<0.05; *n* = 9–13 per group. Statistics display the difference between two mice groups at the indicated time points. Error bars reflect mean ± SEM. (A–G) Data were male mice but similarly observed in females.

Following the studies addressing glucose sensing of hypothalamic HIF (Figures 2–6), we examined whether HIF inhibition in POMC neurons could be sufficient to affect the steady-state levels of feeding and energy homeostasis. First, energy (food) intake and expenditure were profiled in *POMC/HIFβ^lox/lox^* mice and littermate controls under normal chow feeding. Compared to the controls, *POMC/HIFβ^lox/lox^* mice were found hyperphagic (Figure 7A) with impaired energy expenditure (Figure 7B&C), but resulting in only a mild overweight condition (unpublished data). Despite the lack of dramatic body weight effect, DEXA scanning revealed that fat mass of *POMC/HIFβ^lox/lox^* mice increased evidently (Figure 7D). Morphological examination of various fat tissues further confirmed that the size of fat cells isolated from the knockout mice increased (Figure 7E). These physiological changes in the knockout mice were associated with impaired thermogenic response of brown fat tissue to re-feeding (Figure S11). We also tested if the obesogenic effect of hypothalamic HIF loss-of-function could result from leptin resistance. To do this, *ob/ob* mice received mediobasal hypothalamic injection of lentiviruses expressing dominant-negative HIFα, which has been established to inhibit both HIF1α and HIF2α [51],[52]. The results revealed that the obesity-promoting effect of HIF loss-of-function remained in *ob/ob* mice (Figure S12), indicating that leptin signaling was not involved in the metabolic action of hypothalamic HIF. Taken together, HIF loss-of-function in POMC neurons causes positive energy balance in favor of obesity development.

### HIF Inhibition in POMC Neurons Exacerbates Dietary Obesity

To further elucidate the significance of hypothalamic HIF inhibition in obesity development, we maintained *POMC/HIFβ^lox/lox^* mice and the littermate controls under high-fat diet (HFD) feeding since weaning. Despite caloric abundance (58.5 Kcal% fat) in the HFD, *POMC/HIFβ^lox/lox^* mice continued to display an overeating behavior (Figure 7F). Thus, the knockout mice were insensitive to the enriched levels of calories from HFD, again supporting the notion that HIF inactivation in POMC neurons reduced the nutrient-sensing function of the hypothalamus. We monitored the longitudinal course of body weight gain and obesity development in HFD-fed *POMC/HIFβ^lox/lox^* mice versus controls. Compared to HFD-fed control mice, HFD-fed *POMC/HIFβ^lox/lox^* mice gained body weight more rapidly and displayed obesity in an exacerbated manner (Figure 7G). Thus, when challenged with obesity-prone conditions (such as HFD feeding), the susceptibility of disease development is highly increased by HIF dysfunction in hypothalamic POMC neurons, supporting the importance of HIF in the pathogenesis of obesity-diabetes syndrome.

### Treatment of Obesity by Targeting HIF in the Hypothalamus

**Figure 8:**
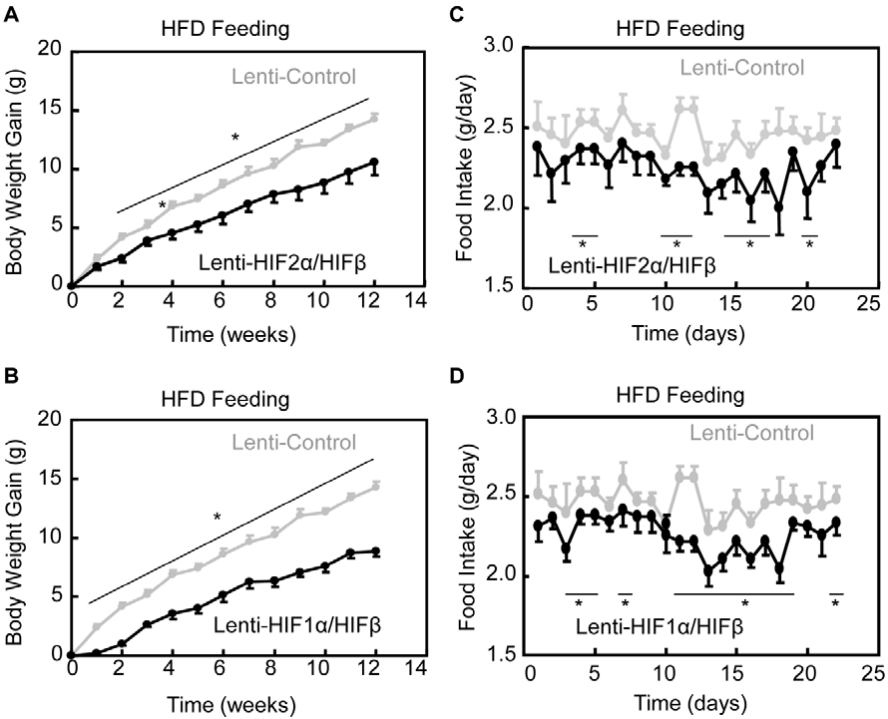
Anti-obesity effect of delivering HIF to the hypothalamus. Age- and weight-matched C57BL/6 mice received mediobasal hypothalamic injection of neuron-specific lentiviruses expressing HIF2α/HIFβ (Lenti-HIF2α/HIFβ) (A&C), HIF1α/HIFβ (Lenti-HIF1α/HIFβ) (B&D), or GFP (Lenti-Control) (A–D). Mice were subsequently maintained under HFD feeding and monitored for body weight gain (A&B) and HFD intake (C&D). The control mice were shared by both groups but repeatedly presented in separate figures. Statistics show the difference between two mice groups at the time points indicated by lines. * *p*<0.05; *n* = 5–8 per group. Error bars reflect mean ± SEM.

Finally, we performed animal studies to evaluate whether hypothalamic HIF could be targeted to generate strong therapeutic effects against obesity. Although HIF2α is the major subunit in the hypothalamus (Figure 1C&D), we designed experiments to evaluate the potential use of both HIF2α and HIF1α. Using the lentiviral co-expression system shown in Figure S10A&B, we delivered HIF2α/HIFβ versus HIF1α/HIFβ into the mediobasal hypothalamus of normal C57BL/6 mice. Matched mice with gene delivery of GFP were used as the controls. Following viral injection, all mice were maintained on an HFD and longitudinally followed up for feeding and weight gain. As shown in Figure 8A&B, the control mice gained body weight, rapidly leading to overt obesity over a 3-mo period. In contrast, delivery of either HIF2α/HIFβ or HIF1α/HIFβ markedly reduced obesity development (Figure 8A&B). The anti-obesity effect of HIF gain-of-function was clearly attributed to feeding restriction (Figure 8C&D) presumably resulting from POMC gene expression up-regulation (Figure S10D). In conclusion, although hypothalamic HIF2α/HIFβ is more physiologically relevant to metabolic regulation, both HIF2α and HIF1α hold significant therapeutic potentials and can be targeted, individually or in combination, in order to counteract obesity and related metabolic diseases.

## Discussion

**Figure 9:**
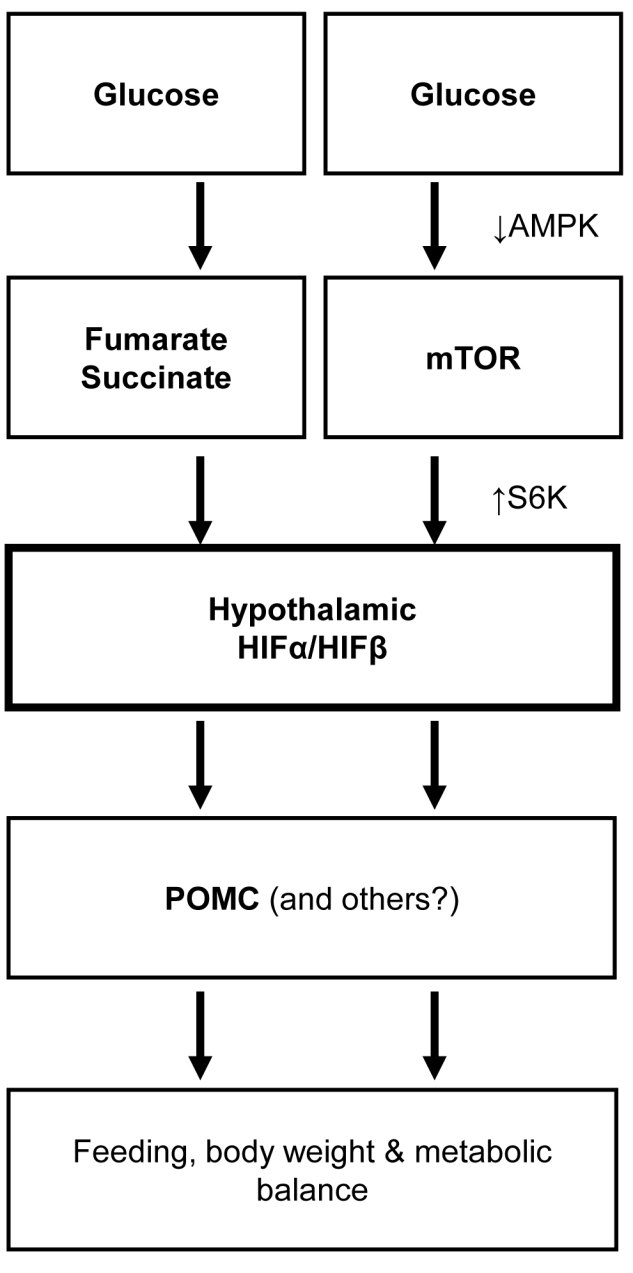
Glucose sensing of HIF in hypothalamic control energy and metabolic balance. Proposed model of hypothalamic HIF in neural control of energy balance. Glucose activates hypothalamic HIF through metabolite-induced suppression of HIF protein degradation and AMPK/mTOR-related promotion of HIF protein synthesis. Activation of HIF up-regulates POMC gene, linking hypothalamic glucose sensing to the hypothalamic control of feeding, energy expenditure, body weight, and metabolic homeostasis. In conclusion, HIF in the hypothalamus represents a molecular target of protecting hypothalamic nutrient sensing in order to improve whole-body metabolic regulation of the hypothalamus and prevent against obesity and related diseases.

This study demonstrates that HIF is present in the hypothalamus and sensitively up-regulated by the local availability of glucose and its metabolites. Glucose up-regulation of hypothalamic HIF is mediated by PHD/pVHL-dependent HIFα degradation and AMPK/mTOR-dependent HIFα synthesis. POMC neurons in the hypothalamus are critical for the metabolic role of hypothalamic HIF through its direct transcriptional regulation on *POMC* gene. HIF loss-of-function in POMC neurons can cause overeating and weight gain to promote obesity development, while HIF gain-of-function can provide strong therapeutic benefits against obesity (and related diseases) (Figure 9). Taken together, all these unexpected findings reveal an unappreciated role for neuronal HIF in the brain regulation of energy, body weight, and metabolic balance.

Energy homeostasis is fundamental for living, and this physiological process relies on the complex property of the life' regulatory system in coordinately sensing and transducing the metabolic dynamics in the body. The hypothalamus is known as the “headquarters” for the regulation of energy balance [17],[18],[53],[54]. This work discovered that the HIF complex plays a critical role in linking hypothalamic glucose sensing to glucose-dependent hypothalamic control of feeding and energy balance. Known as a nuclear transcription factor that mediates hypoxia adaptation, the physiological and disease significance of HIF has traditionally been explored for the pathological roles in diseases, particularly cancers [1]–[3]. Recently, research in cancer biology has begun to appreciate that tumorigenesis involves the connection between HIF and metabolism at cellular levels [55],[56]. Here, we have demonstrated an important but unappreciated role of HIF in the hypothalamus at an organism level from the perspective of feeding and body weight regulation as well as metabolic disease, especially obesity and related health problems. Indicated by our findings, further lines of research can be expected to explore possible roles of neuronal HIF in other physiological functions and disease relevance of the hypothalamus.

For almost a decade, the hypothalamus has been established as a lipostatic feeding regulator through hormonal action of leptin, which involves activation of POMC by STAT3 [37]–[39],[57]. Recently, the role of the hypothalamus in nutrient sensing has become a major interest in the research field, leading to the elucidation of hypothalamic glucose sensing involving AMPK [30]–[32] and KATP channels [27] and hypothalamic amino acid-sensing process involving mTOR [24]. In this study, we discovered that HIF in the hypothalamus acts as a nutrient sensor. In alignment with the fast turnover rate of HIF proteins (half life: 5∼10 min) [1],[2], the HIF pathway in hypothalamic POMC neurons likely directs the real-time homeostasis of feeding and energy homeostasis. This regulation complements STAT3-dependent *POMC* gene expression in leptin signaling and its long-term regulation on feeding and body weight [39],[57],[58]. Additional research seems needed to address if HIF pathway can work in other neuronal types in addition to POMC neurons in mediating hypothalamic control of metabolic physiology. Overall, this work has specifically focused on the glucose-sensing process of the hypothalamus, and along this line, it deserves further endeavors to study whether HIF pathway can function in the central sensing process of other nutrient species (such as amino acids and fatty acids) and their metabolites.

The hypothalamus has been well appreciated as the master regulator of body weight and metabolic balance and a pathogenic culprit for obesity and related disease [17]–[21]. Recent research attentions have been extensively directed to hypothalamic hormonal dysregulations and most notably the development of leptin resistance and insulin resistance [59]–[63]. However, it remains poorly understood how hypothalamic nutrient sensing is mediated. More importantly, it is still unclear if a molecular pathway in central nutrient sensing could be targeted to effectively counteract obesity and related disease. In this work, we found that enhancing hypothalamic glucose sensing through HIF induction per se is effective in treating obesity. Our findings are supported by a recent work showing an anti-obesity effect of HIF activation through the ablation of an HIF inhibitor FIH throughout the brain [64]. We recognize that there is a great deal of research efforts aimed to develop HIF inhibitors for cancer therapeutics, but concerns were recently raised about some serious problems that can arise from HIF inhibition in certain tissues and cells [65]. Here our research further points out that HIF inhibition in the hypothalamus can result in adverse metabolic outcomes and needs to be avoided in drug designs. On the other hand, selective activation of neuronal HIF especially in the hypothalamus could be developed to provide a new therapeutic avenue against obesity and related metabolic disease. Neuron-specific HIF activation might not have critical concerns in terms of oncogenesis since neurons are non-replicable, but the potential application of this strategy in treating metabolic disease will certainly require future technology development and safety assessment.

## Materials and Methods

### Animal Models and Metabolic Phenotyping

*HIFβ^lox/lox^* mice [41] and *POMC-Cre* mice [40] described previously were maintained on the C57BL/6 strain background. *ROSA-flox-STOP-flox-YFP* mice were from Jackson Laboratory. All the mice were housed in standard conditions. High-fat diet (58.5 Kcal% fat) was purchased from Research Diets, Inc. Mice were measured for body weight and food intake on either a daily or regular basis. DEXA scanning was performed using the DEXA scanner at the Primate Center at the University of Wisconsin. The physiological markers of energy expenditure, including O_2_ consumption and CO_2_ production, were measured using the metabolic chambers (Columbus Instrument, Inc.) at the DRTC core at Albert Einstein College of Medicine. The Institutional Animal Care and Use Committee approved all the procedures.

### Third Ventricle Cannulation, Chemical Infusion, and Injection

As previously described [59], an ultra-precise small animal stereotactic apparatus (10-µm resolution, David Kopf Instrument) was used to implant a guide cannula into the third ventricle of anesthetized mice at the midline coordinates of 1.8 mm posterior to the bregma and 5.0 mm below the bregma. The mice were allowed 2 wk for surgical recovery. Angiotensin II-stimulated drink response was used to verify success of implantation. Individual mice were restrained in a mouse restrainer, and infused with an indicated reagent over the indicated time period a 26-gauge guide cannula and a 33-gauge injector (Plastics One) connected to a Hamilton Syringe and an infusion pump (Harvard Apparatus). Glucose, diethyl fumarate ester, and diethyl succinate ester were from Sigma. Nutrient/chemical infusion experiments for terminal molecular assays: glucose (10 nmol/min), diethyl fumarate (50 nmol/min), and diethyl succinate (50 nmol/min) were infused over 2 to 8 h. Rapamycin and AICAR injections were, respectively, 50 ng and 15 µg per injection (at hour 0, 2, and 4 during the 5-h experimental period). The injection experiments for acute physiological tests used a single injection of glucose (40 nmol), diethyl fumarate ester (0.2 µmol), diethyl succinate ester (0.2 µmol), and AICAR (3 µg) in a 2 µl vehicle over 5 min.

### Chemicals, Plasmids, and Recombinant Lentiviral Vectors

Glucose and rapamycin were from Sigma, and AICAR from Toronto Research Chemicals. Artificial cerebrospinal fluid (aCSF) was used as the vehicle for glucose, leucine, insulin, leptin, and AICAR. Full-length cDNAs for HIF1α, HIF2α, and HIFβ, provided by D. Peet, were sub-cloned into pcDNA3.1 plasmids (Invitrogen). DNA for POMC promoter (provided by S. Melmed) was sub-cloned to pGL3 (Promega). Mutant POMC promoter was generated by deleting 5′-GCGTG-3′ in the WT version of POMC promoter. For the lentivirus that co-expresses HIF subunits, we introduced the encoding cDNA of HIF1α or HIF2α and HIFβ into the lentiviral vector Lox-Syn-Syn (provided by G. Francisco) in which two synapsin promoters control neuron-specific co-expression of two inserts. For lentivirus that directs expression of single gene, we sub-cloned PCR fragment of dominant-negative HIF2α (HIF2α amino acids 1–485) [51],[52] or constitutively active Rheb (^CA^Rheb) (provided by J. Avruch) into pLenti6/V5 vector (Invitrogen).

### Viruses, Injection, and Verification

The lentiviruses were produced from HEK293T cells through co-transfecting the target plasmid with two package plasmids (VSVg and delta 8.9) using Ca_3_(PO_4_)_2_. Lentiviruses were purified by ultracentrifugation. Ultracentrifuge purified lentivirus in 0.2 µl aCSF was injected over 10 min through a 26-gauge guide cannula and a 33-gauge injector (Plastics One) connected to a Hamilton Syringe and an infusion pump (WPI Instruments). As previously described [59], bilateral injections to the mediobasal hypothalamus were directed using an ultra-precise stereotax with 10-µm resolution (Kopf Instruments) to the coordinates of 1.5 mm posterior to the bregma, 5.8 mm below the bregma, and 0.3 mm lateral to midline.

### Western Blots and Histology

As we previously described [59], the hypothalamus was dissected along the anterior border of the optic chiasm, posterior border of the mammillary body, upper border of the anterior commissure, and lateral border halfway from the lateral sulcus in the ventral side of brain. Animal tissues were homogenized, the proteins dissolved in a lysis buffer, and Western blot was performed as previously described [59],[66],[67]. Proteins dissolved in a lysis buffer were separated by SDS/PAGE and identified by immunoblotting or immunoprecipitation followed by immunoblotting. Primary antibodies included anti-HIF1α, anti-HIF2α, and anti-PHD2 (Novus Biologicals); anti-p300 (Santa Cruz); and anti-VHL, anti-pS6K, anti-S6K, anti-pAMPKα, anti-AMPKα, and anti-β-actin (Cell Signaling). Secondary antibodies included HRP-conjugated anti-rabbit and anti-mouse antibodies (Pierce). Western blots were quantified using NIH Image J software. Tissue histology: Various tissues were removed from mice and fixed in Bouin' solution (Sigma). Parafilm sections were prepared, stained with H&E, and examined under a bright field microscope.

### GHO Assay and Biochemical Measurements

PHD activity was determined using the GHO assay as established in the literature [42]. Briefly, a wheat germ in vitro transcription-translation (IVTT) system (Promega) was used to produce unhydroxylated HA-tagged GHO protein (substrate of PHDs). Hydroxylation of GHO protein was performed by incubation with hypothalamic protein lysates (dissolved in HEB buffer) at 37°C for 15 min in the presence of 1 mM ascorbate and 100 µM FeSO_4_ (Sigma). The reaction was terminated by adding SDS loading buffer, and hydroxylated versus unhydroxylated GHO protein was separated by PAGE gels and detected by Western blot analysis of HA tag. Fumarate and succinate were measured using Fumarate Assay Kit (BioVison) and Succinic Acid Kit (Megazyme).

### Cell Culture and Luciferase Assay

HEK 293 and HEK 293T (ATCC) were maintained in DMEM with 5%–10% FBS, glutamate, antibiotics, and in 5%–10% CO_2_ at 37°C. Transfection of cultured cells with luciferase plasmids and expression plasmids was performed through Lipofectamine 2000 (Invitrogen). Co-transfection of pRL-TK (Promega) was used to internally control firefly activity. Empty plasmids pGL3 and pcDNA3.1 were used as negative controls.

### Quantitative RT-PCR

We extracted total RNA from the homogenized hypothalamus using TRIzol (Invitrogen). Complementary DNA was synthesized using the M-MLV RT system (Promega). PCR amplification was quantified using SYBR®Green PCR Master Mix (Applied Biosystems). Results were normalized against the expression of house-keeping genes including TATA box-binding protein (TBP) and GAPDH.

### Heart Perfusion, Immunostaining, and Imaging

Mice under anesthesia were trans-heart perfused with 4% PFA, and the brains were removed, post-fixed in 4% PFA for 4 h, and infiltrated in 20%–30% sucrose. Brain sections (20-µm thickness) were made using a cryostat at −20°C. For cell culture, cells were cultured in coverslips and fixed using 4% PFA. Fixed tissues or cells were blocked with serum of appropriate species, penetrated with 0.2% Triton-X 100, treated with primary antibodies including rabbit anti-HIF1α, anti-HIF2α (Novus Biologicals), anti-HIFβ (Cell Signaling), mouse anti-HuCD (Molecular Probes), and subsequently followed by a fluorescent reaction with Alexa Fluor 488 or 555 secondary antibody (Invitrogen). Naïve IgGs of the appropriate species were used as negative controls. DAPI staining was used to reveal all the cells in the slides. A con-focal microscope was used to image fluorescence.

### Behavioral Tests and Treatments

#### Fasting induced food intake

The individually housed mice were measured for food intake. One hour before the designated “night time,” a reagent (nutrient species) dissolved in 1–2 µl of vehicle or the empty vehicle (control) was injected to fasted mice through third ventricle cannula that were pre-implanted 1 to 2 wk prior to the commencing of experiments. Food was then returned to the mice immediately before the designated “night time.” Food intake for each mouse was measured for the following periods as indicated. *Hypoxia treatment*: Animal was maintained in a hypoxia chamber supplied with 8% O_2_ and 92% nitrogen for 6 h as established in the literature [68].

#### Statistical analyses

ANOVA and Tukey' post hoc analyses were used for comparisons, which involved more than two groups. Kolmogorov-Smimov (KS) test was applied to each dataset to determine the appropriate statistical test (e.g., parametric or nonparametric) for analysis of each set of data. Two-tailed Student' *t* tests were used for comparisons, which involved only two groups. Data from electrophysiology recording were compared using paired *t* test. Software for performing statistics included Excel, GraphPad Instat 3, and Prism. Data were presented as mean ± SEM. *p*<0.05 was considered significant.

## Supporting Information

Figure S1 Verification of HIF2α antibody specificity. Hypothalamic GT1-7 cells cultured in slides were transfected with pcDNA3.1 vector expressing myc-tagged HIF2α (left panel), myc-tagged HIF1α (right panel), or empty vector (middle panel). Cells were fixed and co-immunostained using HIF2α antibody (red) and myc antibody (green). Nuclei of all cells in the slides were revealed by DAPI staining (blue). Merge of colors indicates co-immunostaining by the two antibodies. Bar = 50 µm.
(TIF)

Figure S2 Verification of HIF1α antibody specificity. Hypothalamic GT1-7 cells cultured in slides were transfected with pcDNA3.1 vector expressing myc-tagged HIF1α (left panel), myc-tagged HIF2α (right panel), or empty vector (middle panel). Cells were fixed and co-immunostained using HIF1α antibody (red) and myc antibody (green). Nuclei of all cells were revealed by DAPI staining (blue). Merge of colors indicates co-immunostaining by the two antibodies. Bar = 50 µm.
(TIF)

Figure S3 Verification of HIFβ antibody specificity. Hypothalamic GT1-7 cells cultured in slides were transfected with pcDNA3.1 vector expressing HA-tagged HIFβ. Cells were fixed and co-immunostained using anti-HIFβ antibody (red) and anti-HA antibody (green). Nuclei of all cells were revealed by DAPI staining (blue). Merge of colors indicates co-immunostaining by the two antibodies. Bar = 50 µm.
(TIF)

Figure S4 Gene expression profiles in POMC/HIFβ^lox/lox^ mice. (A&B) Following 24-h fasting, *POMC/HIFβ^lox/lox^* mice (*P/HIFβ^l/l^*) and control *HIFβ^lox/lox^* mice (*HIFβ^l/l^*) received 6-h re-feeding (R) versus continued 6-h fasting (F). Hindbrain (A) and hypothalami (B) were subsequently harvested for the measurement of mRNA levels of indicated genes. *n* = 6–8 per group. Error bars reflect mean ± SEM. (C) Following 24-h fasting, C57BL/6 mice received injection of glucose versus vehicle via cannula pre-implanted into the third ventricle. Hypothalami were harvested for the measurement of HIF1α versus HIF2α mRNA levels. *n* = 5–8 per group. Error bars reflect mean ± SEM.
(TIF)

Figure S5 Profile of pituitary POMC-ATCH system in POMC/HIFβ^lox/lox^ mice. (A–D) *POMC/HIFβ^lox/lox^* (*P/HIFβ^lox/lox^*) mice and littermate control *HIFβ^lox/lox^* mice were analyzed for pituitary ACTH immunostaining (A&B), blood ACTH concentration (C), and pituitary weight (D). (A) Pituitary sections contained anterior pituitary (AP) and posterior pituitary (PP) that had no ACTH cells and was outlined by broken lines. (B) ACTH cells were counted based on pituitary cross-sections that were cut at midline point, and data presented represent the analysis of at least 3 mice per group. Error bars reflect mean ± SEM. (E&F) *POMC/HIFβ^lox/lox^* (*P/HIFβ^lox/lox^*) mice and littermate controls (*HIFβ^lox/lox^* mice) were analyzed for adrenal gland morphology via H&E staining (E) and adrenal gland weight (F). (G) *POMC/HIFβ^lox/lox^* (*P/HIFβ^lox/lox^*) mice and control littermates (*HIFβ^lox/lox^* mice) mice were psychosocially stressed or intact. Serum samples were collected from these mice and measured for corticosterone concentrations. *n* = 5–6 per group. Error bars reflect mean ± SEM.
(TIF)

Figure S6 Profiles of hypothalamic versus peripheral glucose-HIF connection. (A) Following 24-h fasting, C57BL/6 mice received third-ventricle injection of glucose at the indicated doses. HIF2α protein levels in the hypothalamus were examined by Western blots. β-actin was used as an internal control. (B&C) Following 24-h fasting, C57BL/6 mice received intraperitoneal injection of glucose (Glu) (2 g/kg body weight) or vehicle. HIF2α protein levels in the liver (B) and lung (C) tissues were examined by Western blots. β-actin was used as an internal control.
(TIF)

Figure S7 Relationship between leptin signaling and hypothalamic HIF pathway. (A) C57BL/6 mice received third ventricle injection of leptin or vehicle (Veh). The hypothalami were harvested for Western blot analysis HIF2α protein levels. β-actin was used as an internal control. (B) Regular C57BL/6 mice received third ventricle infusion of glucose (Glu), leucine (Leu), or vehicle (Veh). For comparison, a subset of mice received third ventricle injection of leptin (Lep). Hypothalami were harvested for Western blot analysis of STAT3 phosphorylation (pSTAT3) and STAT3 protein levels. Western blots of β-actin were performed as an internal control. (C&D) *POMC/HIFβ^lox/lox^* mice (*P/HIFβ^lox/lox^*) and control littermate *HIFβ^lox/lox^* mice received third ventricle injection of leptin or control vehicle. Brain sections of mediobasal hypothalamus were prepared and immunostained for phosphorylated STAT3 (pSTAT3) (green) and α-MSH (red). DAPI nuclear staining (blue) revealed all cells in the sections. ARC, arcuate nucleus. Bar = 50 µm. (D) POMC neurons (α-MSH-immunoreactive) positive for pSTAT3 in multiple sections were counted and analyzed statistically. Data represent the observations from at least 3 mice per group. Error bars reflect mean ± SEM. (E) Young, male *POMC/HIFβ^lox/lox^* mice (*P/HIFβ^lox/lox^* mice) versus littermate control *HIFβ^lox/lox^* mice were fasted 24 h and received third-ventricle injection of leptin or vehicle. Food was placed in cages, and mice were subsequently monitored for food intake. * *p*<0.05; *n* = 6–8 per group. Error bars reflect mean ± SEM.
(TIF)

Figure S8 Dose-course and time-course actions of fumarate or succinate. Following 24-h fasting, C57BL/6 mice received third-ventricle infusion of diethyl fumarate or diethyl succinate at the dose of 0, 0.6, 3.0, or 15 µmol/h for 5 h (A&C) or at the dose of 3.0 µmol/h for 0, 2, 5, or 8 h (B&D). Hypothalami were harvested and measured for the tissue contents of fumarate and succinate (A–D) and HIF2α protein (E&F). Bar graphs: * *p*<0.05; *n* = 6–8 per group. Error bars reflect mean ± SEM.
(TIF)

Figure S9 Food intake and taste aversion effects of pharmacologic chemicals. (A) Following 24-h fasting, C57BL/6 mice received third-ventricle injection of trans-aconitate (ACON), thenoyltrifluoroacetone (TTFA), 3-nitropropionic acid (3NPA), or vehicle (Veh). Food was provided to mice and food intake of mice was recorded. * *p*<0.05; *n* = 7–10 per group. Error bars reflect mean ± SEM. (B) C57BL/6 mice were habituated to experimental protocol for several days and then presented to 0.2% saccharine for 60 min after removing the drinking water for 23 h. After habitation, mice were ICV injected with an indicated drug and the vehicle (Veh) via pre-implanted third ventricle cannula, and subsequently had access to 0.2% saccharine for 60 min (Test 1). Intraperitoneal (IP) injection of LiCl and saline was used as a positive and negative control, respectively. After 4 d, mice were presented with 0.2% saccharine, and saccharine intake of mice during 60 min was measured (Test 2). Data represent 60-min saccharine intake in Test 2. ** *p*<0.01, *ns*, non-significant; *n* = 6–7 per group. Error bars reflect mean ± SEM. ACON, trans-aconitate; TTFA, thenoyltrifluoroacetone; 3NPA, 3-nitropropionic acid.
(TIF)

Figure S10 Lentivirus-directed hypothalamic HIF delivery up-regulates POMC gene. (A) Schematic map of lentivirus that co-expressed myc-conjugated HIFα (either HIF2α or HIF1α) and HA-conjugated HIFβ (Lenti-HIF2α/HIFβ) under the control of neuron-specific synapsin (Syn) promoter. Matched GFP-expressing lentiviral vector (Lenti-GFP) was used as a control. (B&C) C57BL/6 mice received intra-mediobasal hypothalamic injection of Lenti-HIFα/HIFβ or control Lenti-GFP. (B) Site-specific gene delivery was verified by GFP (green) and HA staining (red). DAPI nuclear staining (blue) reveals all cells in the sections. Right panels: GFP (green) and neuronal marker HuCD staining (red) are merged to indicate neuron-specific gene delivery (yellow). (C) Site-specific gene delivery was verified by Western blot analysis of myc and HA expression. Data shown in (B&C) were obtained from the mice injected with Lenti-HIF2α/HIFβ versus Lenti-GFP, but also represented similar patterns in mice injected with Lenti-HIF2α/HIFβ versus Lenti-GFP. ARC, arcuate nucleus; 3V, third ventricle; β-act, β-actin. Bar = 50 µm. (D) Chow-fed regular C57BL/6 mice that received bilateral MBH injections of Lenti-HIF2α/HIFβ or Lenti-GFP. At 2 wk post-injection, hypothalami were harvested for the measurement of POMC mRNA levels. * *p*<0.05; *n* = 5 per group. Error bars reflect mean ± SEM.
(TIF)

Figure S11 Thermogenic activities in POMC/HIFβ^lox/lox^ mice. Following 24-h fasting, *POMC/HIFβ^lox/lox^* mice (*P/HIFβ^l/l^*) and control *HIFβ^lox/lox^* mice (*HIFβ^l/l^*) received 6-h re-feeding versus continued 6-h fasting. Hypothalami were collected and analyzed for mRNA levels of indicated genes. * *p*<0.05, ** *p*<0.01, *ns*, non-significant; *n* = 5–8 per group. Error bars reflect mean ± SEM.
(TIF)

Figure S12 Effects of HIF on the metabolic phenotype in ob/ob mice. (A&B) Body weight-matched ob/ob mice (8 wk old) received intra-mediobasal hypothalamic injection of neuron-specific lentiviruses expressing dominant-negative HIF1/2α (^DN^HIF1/2α) or control GFP. Mice were monitored for body weight (BW) gain during 2-wk follow-up (A) and daily food intake during this follow-up period (B). * *p*<0.05; *n* = 5–6 per group. Error bars reflect mean ± SEM.
(TIF)

## Abbreviations

3-NPA: 3-nitropropionic acid
α-MSH: α-melanocyte stimulating hormone
ACTH: adrenocorticotropic hormone
AICAR: Aminoimidazole carboxamide ribonucleotide
AMPK: AMP-activated protein kinase
CNS: central nervous system
GFP: green fluorescent protein
HFD: high-fat diet
HIF: hypoxia-inducible factor
mTOR: mammalian target of rapamycin
PHD: prolyl hydroxylase
POMC: Proopiomelanocortin
pVHL: product of von Hippel-Lindau gene
S6K: S6 kinase
SEM: standard error of the mean
STAT3: signal transducer and activator of transcription 3
TTFA: thenoyltrifluoroacetone
