# Correction: Hypoxia-Inducible Factor Directs POMC Gene to Mediate Hypothalamic Glucose Sensing and Energy Balance Regulation

**DOI:** 10.1371/journal.pbio.1002428

**Published:** 2016-03-25

**Authors:** Hai Zhang, Guo Zhang, Frank J. Gonzalez, Sung-min Park, Dongsheng Cai

It has come to the authors' attention that the HIF2α blot in Fig 6A and the β-actin blot in Fig 3B were inadvertently duplicated in Fig 5A and S6C Fig, respectively. The authors provided original blots and records for the editors to verify and have now reproduced these figures with the correct data. Revised versions of Fig 5 and S6 Fig are provided here.

**Fig 5:**
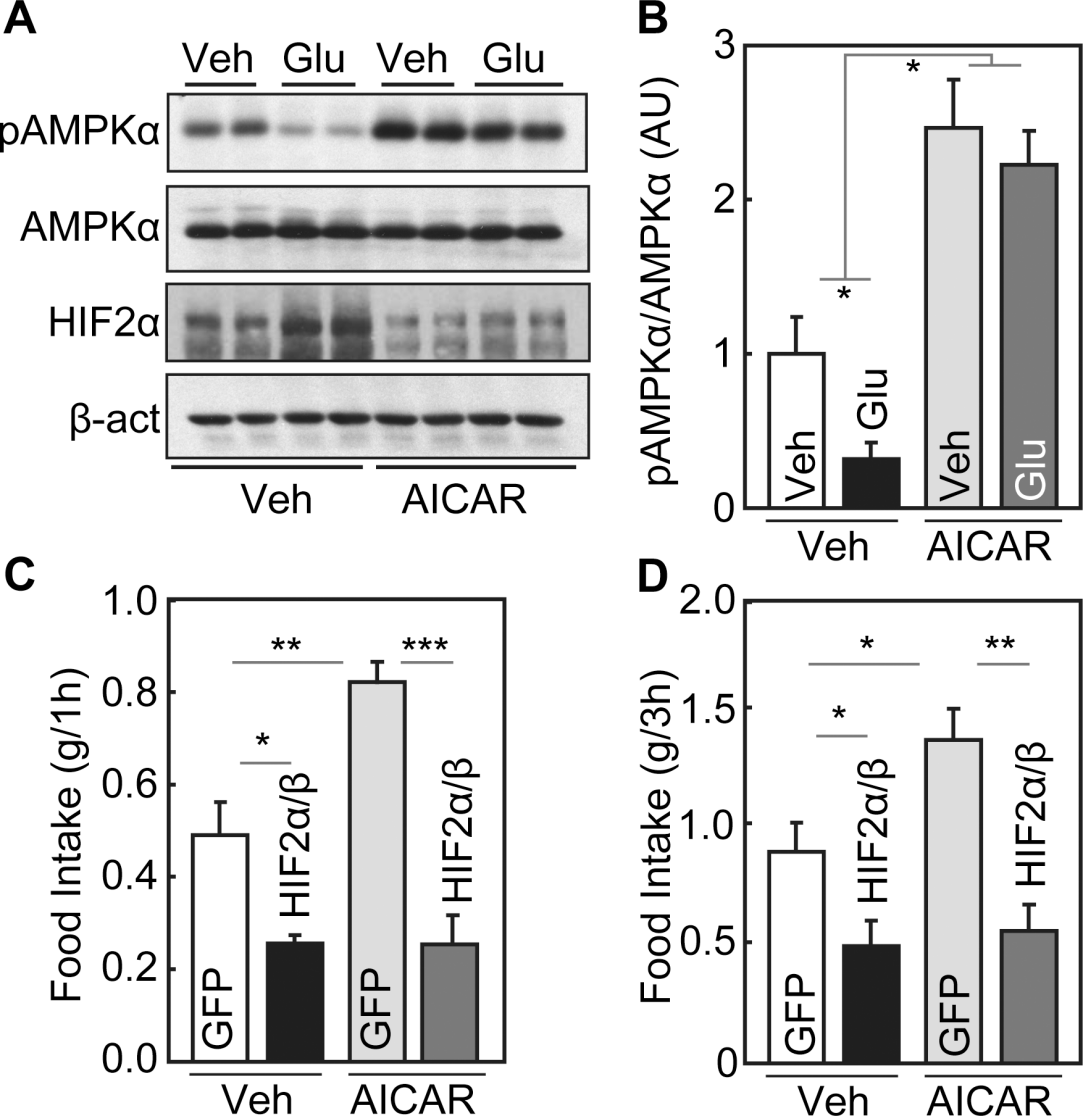
Effect of AMPK in glucose-mediated activation of hypothalamic HIF. (A&B) Western blot analyses of AMPK signaling were performed for the hypothalami harvested from C57BL/6 mice that received a 5-h intra-third ventricle infusion of glucose (Glu) versus vehicle (Veh) in the presence or absence of a prior third-ventricle injection of AICAR. β-act, β-actin. Bar graphs: Western blots were quantitated and analyzed statistically. (C&D) C57BL/6 mice received mediobasal hypothalamic injection of neuron-specific lentiviruses expressing HIF2α/HIFβ (HIF2α/β) or GFP, and simultaneously received cannula implantation into the third ventricles. After 2-wk post-surgical recovery, mice received 6-h fasting and then were injected with AICAR or vehicle (Veh) via the cannula. Mice subsequently had free access to food and were measured for food intake during 1-h (C) and 3-h (D) periods. (B–D) * *p*<0.05, ** *p*<0.01, *** *p*<0.001; *n* = 6 per group. Error bars reflect mean ± SEM.

## Supporting Information

S6 Fig Profiles of hypothalamic versus peripheral glucose-HIF connection. (A) Following 24-h fasting, C57BL/6 mice received third-ventricle injection of glucose at the indicated doses. HIF2α protein levels in the hypothalamus were examined by Western blots. β-actin was used as an internal control. (B&C) Following 24-h fasting, C57BL/6 mice received intraperitoneal injection of glucose (Glu) (2 g/kg body weight) or vehicle. HIF2α protein levels in the liver (B) and lung (C) tissues were examined by Western blots. β-actin was used as an internal control.
(TIF)
