# Supplementary figures and images for: Hypoxia-Inducible Factor Directs POMC Gene to Mediate Hypothalamic Glucose Sensing and Energy Balance Regulation
Source: PLoS Biol. 2011 Jul 26;9(7):e1001112. doi: 10.1371/journal.pbio.1001112 (PMC3144184; doi:10.1371/journal.pbio.1001112)

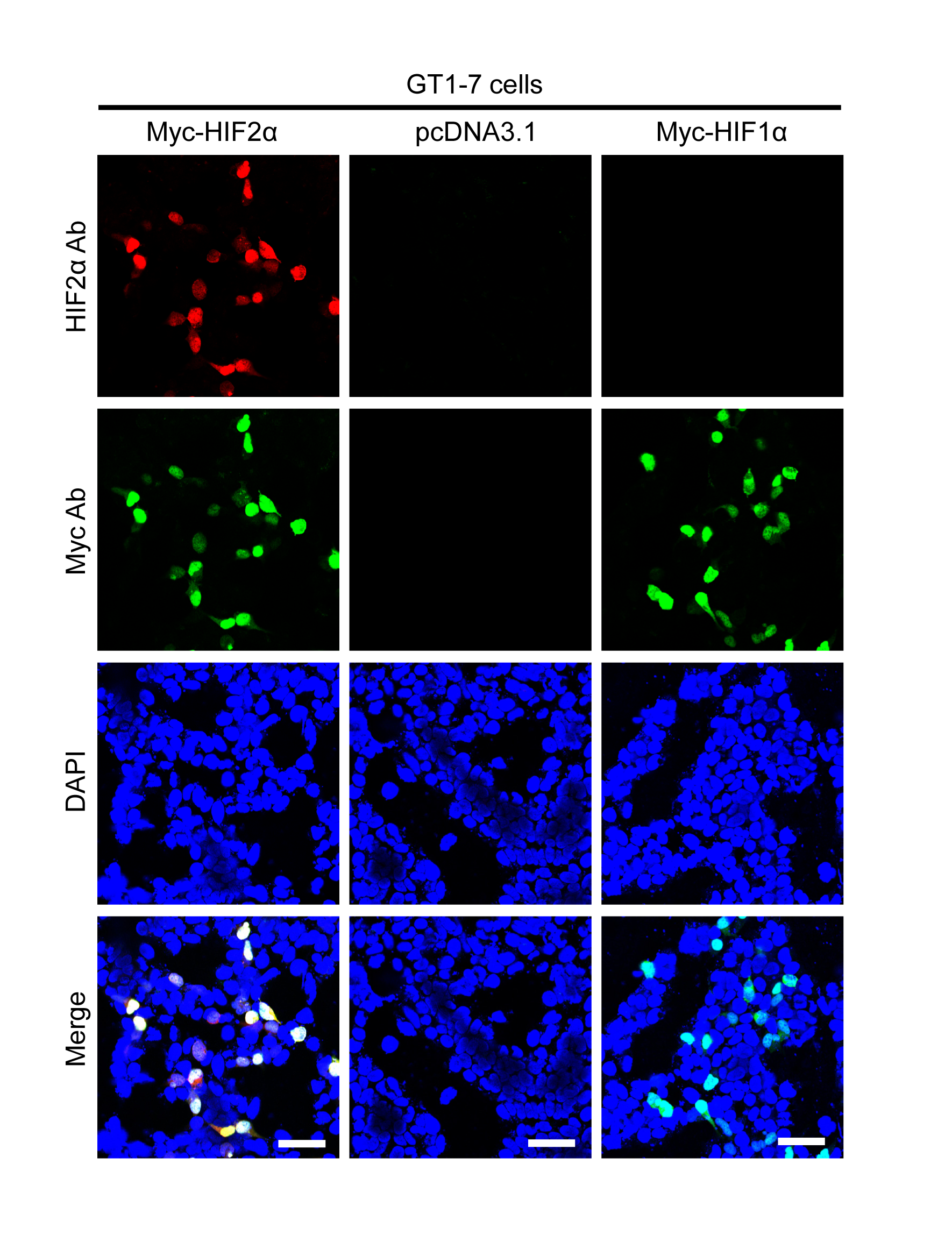

Supplement: Figure S1 — Verification of HIF2α antibody specificity. Hypothalamic GT1-7 cells cultured in slides were transfected with pcDNA3.1 vector expressing myc-tagged HIF2α (left panel), myc-tagged HIF1α (right panel), or empty vector (middle panel). Cells were fixed and co-immunostained using HIF2α antibody (red) and myc antibody (green). Nuclei of all cells in the slides were revealed by DAPI staining (blue). Merge of colors indicates co-immunostaining by the two antibodies. Bar = 50 µm. (TIF) [file pbio.1001112.s001.tif]

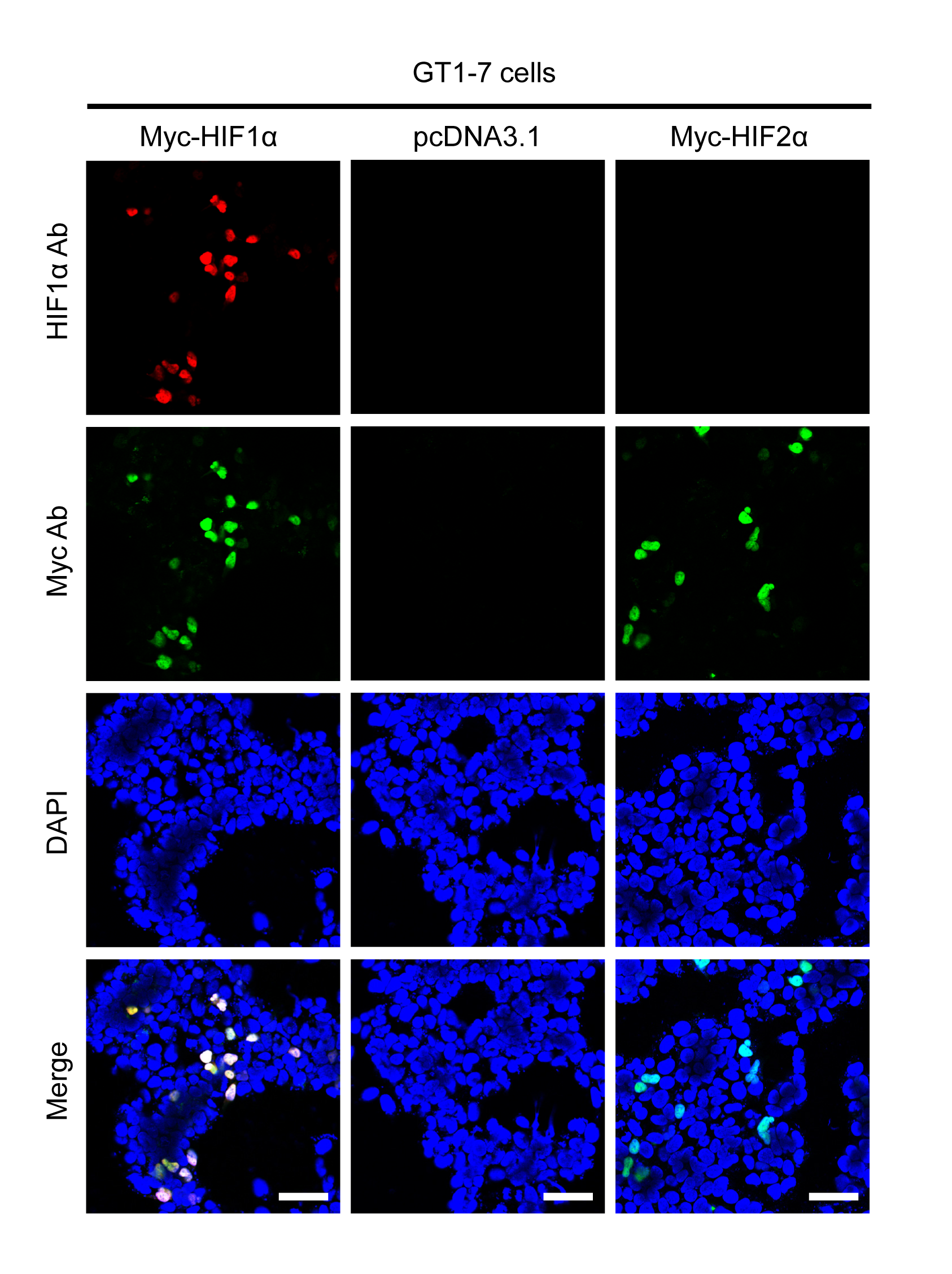

Supplement: Figure S2 — Verification of HIF1α antibody specificity. Hypothalamic GT1-7 cells cultured in slides were transfected with pcDNA3.1 vector expressing myc-tagged HIF1α (left panel), myc-tagged HIF2α (right panel), or empty vector (middle panel). Cells were fixed and co-immunostained using HIF1α antibody (red) and myc antibody (green). Nuclei of all cells were revealed by DAPI staining (blue). Merge of colors indicates co-immunostaining by the two antibodies. Bar = 50 µm. (TIF) [file pbio.1001112.s002.tif]

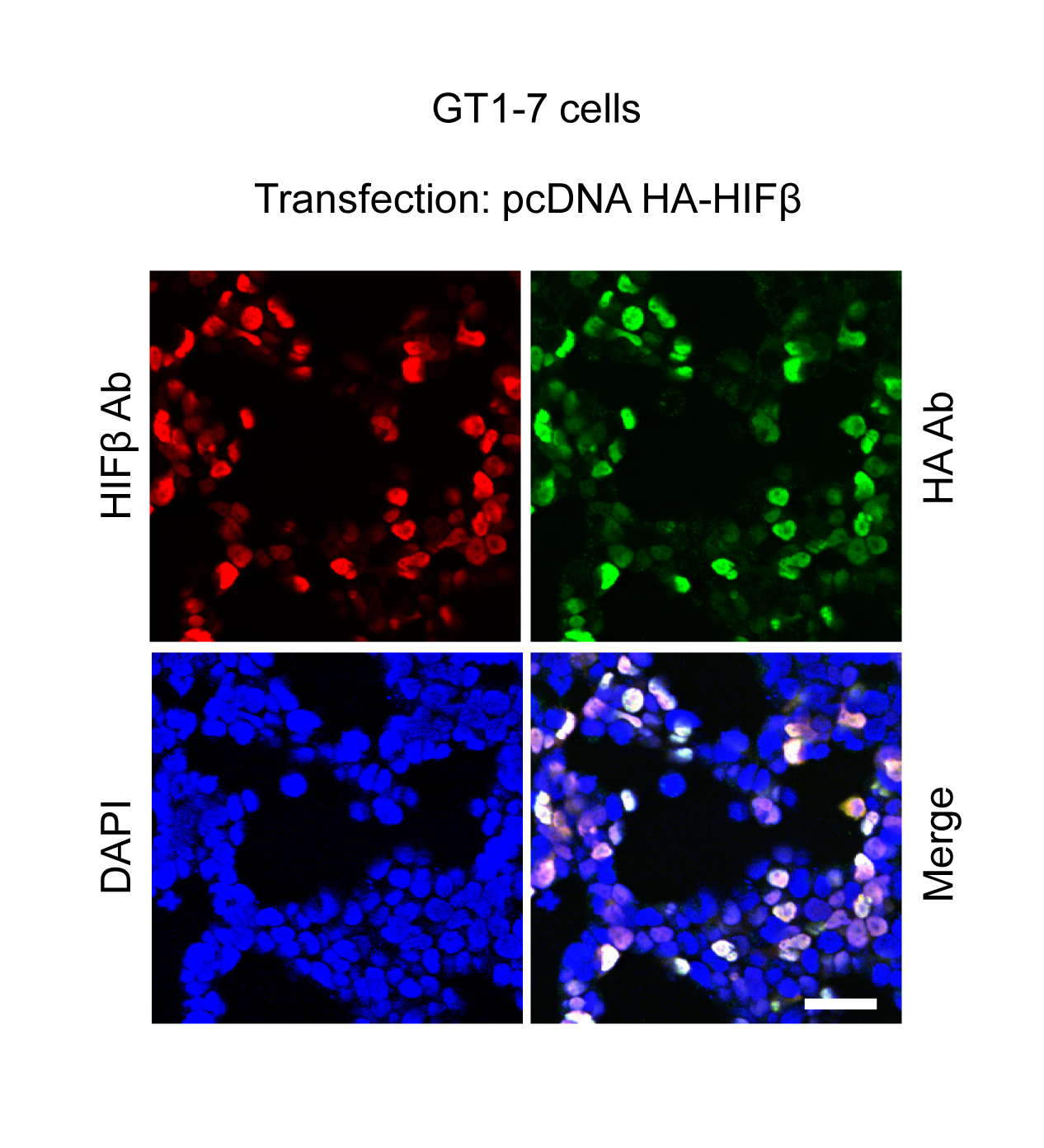

Supplement: Figure S3 — Verification of HIFβ antibody specificity. Hypothalamic GT1-7 cells cultured in slides were transfected with pcDNA3.1 vector expressing HA-tagged HIFβ. Cells were fixed and co-immunostained using anti-HIFβ antibody (red) and anti-HA antibody (green). Nuclei of all cells were revealed by DAPI staining (blue). Merge of colors indicates co-immunostaining by the two antibodies. Bar = 50 µm. (TIF) [file pbio.1001112.s003.tif]

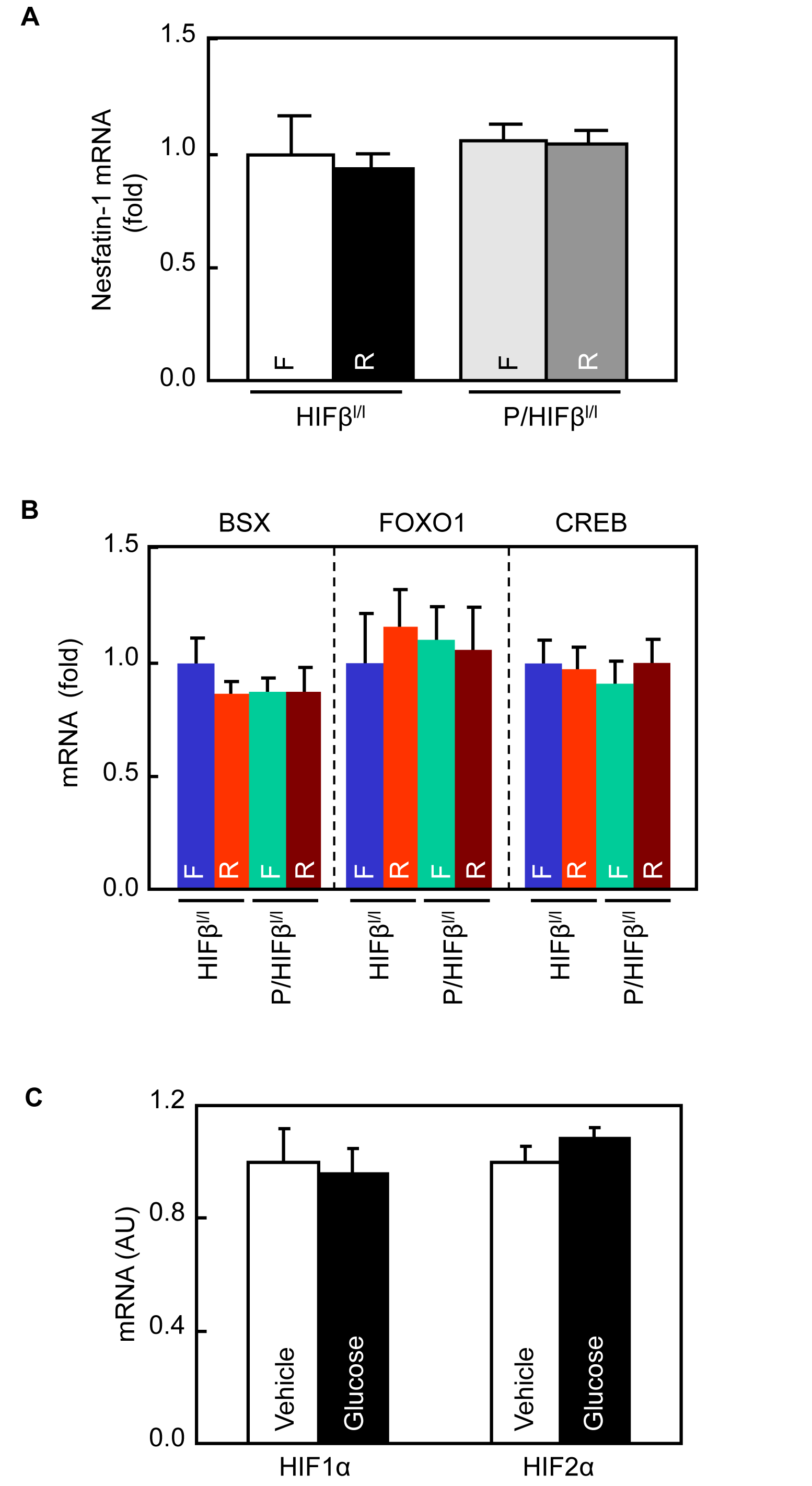

Supplement: Figure S4 — Gene expression profiles in POMC/HIFβlox/lox mice. (A&B) Following 24-h fasting, POMC/HIFβlox/lox mice (P/HIFβl/l) and control HIFβlox/lox mice (HIFβl/l) received 6-h re-feeding (R) versus continued 6-h fasting (F). Hindbrain (A) and hypothalami (B) were subsequently harvested for the measurement of mRNA levels of indicated genes. n = 6–8 per group. Error bars reflect mean ± SEM. (C) Following 24-h fasting, C57BL/6 mice received injection of glucose versus vehicle via cannula pre-implanted into the third ventricle. Hypothalami were harvested for the measurement of HIF1α versus HIF2α mRNA levels. n = 5–8 per group. Error bars reflect mean ± SEM. (TIF) [file pbio.1001112.s004.tif]

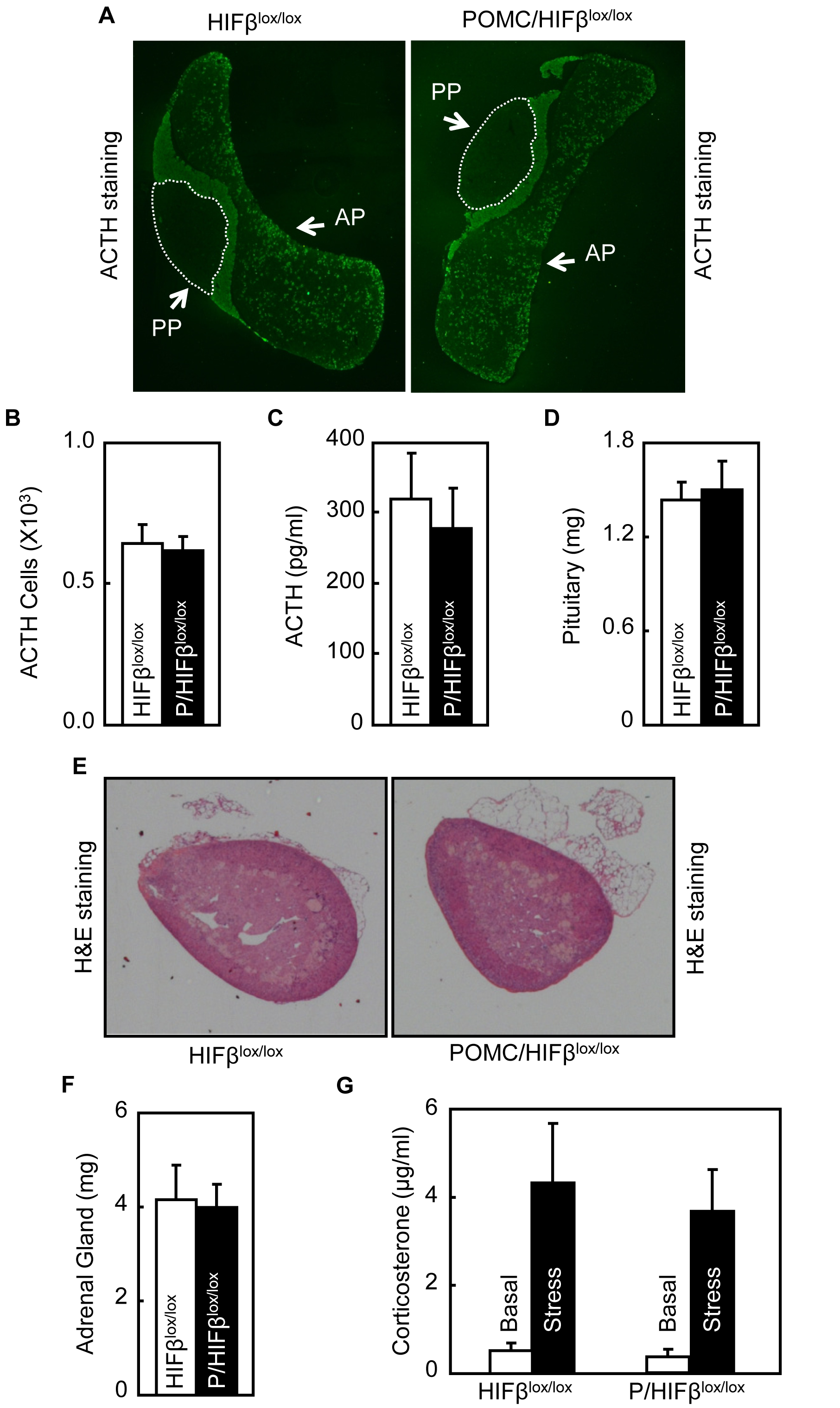

Supplement: Figure S5 — Profile of pituitary POMC-ATCH system in POMC/HIFβlox/lox mice. (A–D) POMC/HIFβlox/lox (P/HIFβlox/lox) mice and littermate control HIFβlox/lox mice were analyzed for pituitary ACTH immunostaining (A&B), blood ACTH concentration (C), and pituitary weight (D). (A) Pituitary sections contained anterior pituitary (AP) and posterior pituitary (PP) that had no ACTH cells and was outlined by broken lines. (B) ACTH cells were counted based on pituitary cross-sections that were cut at midline point, and data presented represent the analysis of at least 3 mice per group. Error bars reflect mean ± SEM. (E&F) POMC/HIFβlox/lox (P/HIFβlox/lox) mice and littermate controls (HIFβlox/lox mice) were analyzed for adrenal gland morphology via H&E staining (E) and adrenal gland weight (F). (G) POMC/HIFβlox/lox (P/HIFβlox/lox) mice and control littermates (HIFβlox/lox mice) mice were psychosocially stressed or intact. Serum samples were collected from these mice and measured for corticosterone concentrations. n = 5–6 per group. Error bars reflect mean ± SEM. (TIF) [file pbio.1001112.s005.tif]

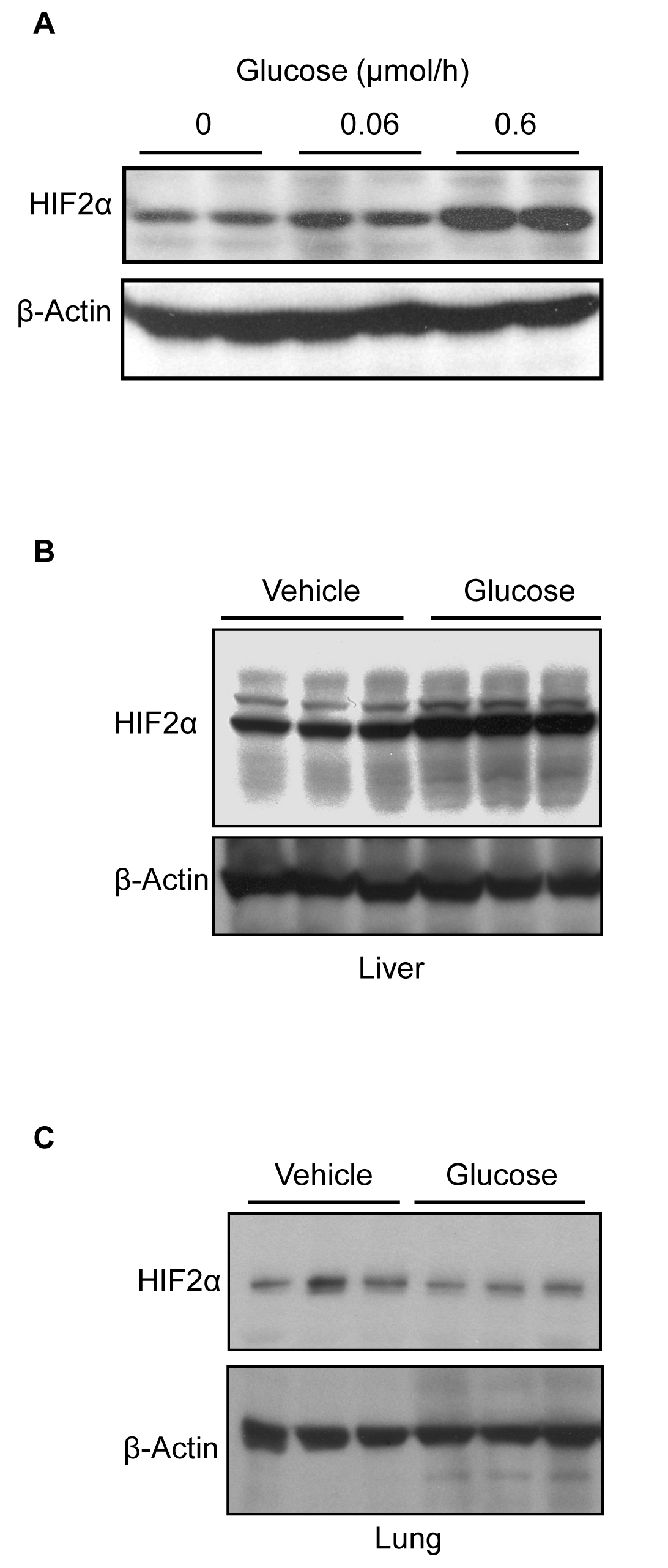

Supplement: Figure S6 — Profiles of hypothalamic versus peripheral glucose-HIF connection. (A) Following 24-h fasting, C57BL/6 mice received third-ventricle injection of glucose at the indicated doses. HIF2α protein levels in the hypothalamus were examined by Western blots. β-actin was used as an internal control. (B&C) Following 24-h fasting, C57BL/6 mice received intraperitoneal injection of glucose (Glu) (2 g/kg body weight) or vehicle. HIF2α protein levels in the liver (B) and lung (C) tissues were examined by Western blots. β-actin was used as an internal control. (TIF) [file pbio.1001112.s006.tif]

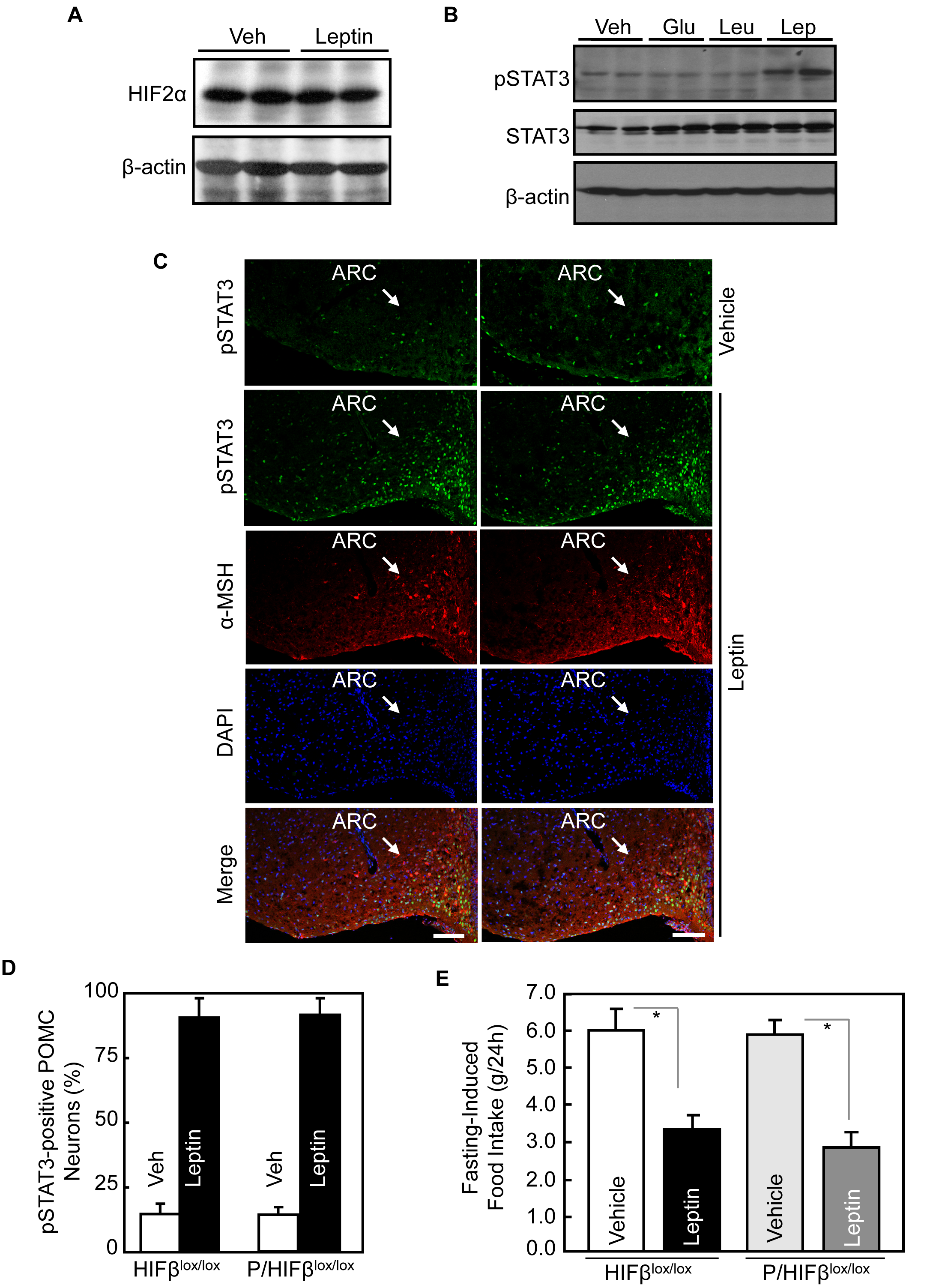

Supplement: Figure S7 — Relationship between leptin signaling and hypothalamic HIF pathway. (A) C57BL/6 mice received third ventricle injection of leptin or vehicle (Veh). The hypothalami were harvested for Western blot analysis HIF2α protein levels. β-actin was used as an internal control. (B) Regular C57BL/6 mice received third ventricle infusion of glucose (Glu), leucine (Leu), or vehicle (Veh). For comparison, a subset of mice received third ventricle injection of leptin (Lep). Hypothalami were harvested for Western blot analysis of STAT3 phosphorylation (pSTAT3) and STAT3 protein levels. Western blots of β-actin were performed as an internal control. (C&D) POMC/HIFβlox/lox mice (P/HIFβlox/lox) and control littermate HIFβlox/lox mice received third ventricle injection of leptin or control vehicle. Brain sections of mediobasal hypothalamus were prepared and immunostained for phosphorylated STAT3 (pSTAT3) (green) and α-MSH (red). DAPI nuclear staining (blue) revealed all cells in the sections. ARC, arcuate nucleus. Bar = 50 µm. (D) POMC neurons (α-MSH-immunoreactive) positive for pSTAT3 in multiple sections were counted and analyzed statistically. Data represent the observations from at least 3 mice per group. Error bars reflect mean ± SEM. (E) Young, male POMC/HIFβlox/lox mice (P/HIFβlox/lox mice) versus littermate control HIFβlox/lox mice were fasted 24 h and received third-ventricle injection of leptin or vehicle. Food was placed in cages, and mice were subsequently monitored for food intake. * p<0.05; n = 6–8 per group. Error bars reflect mean ± SEM. (TIF) [file pbio.1001112.s007.tif]

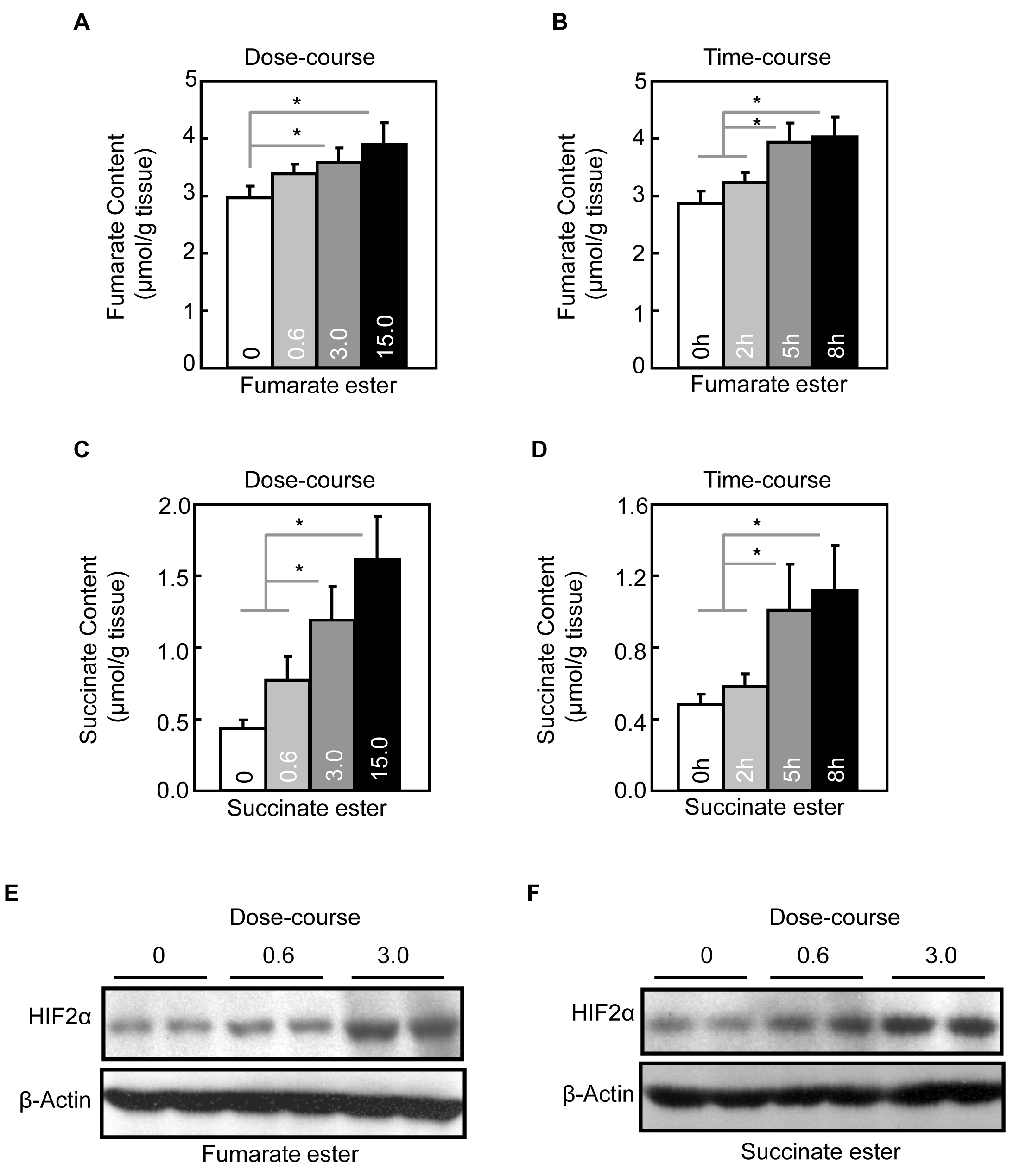

Supplement: Figure S8 — Dose-course and time-course actions of fumarate or succinate. Following 24-h fasting, C57BL/6 mice received third-ventricle infusion of diethyl fumarate or diethyl succinate at the dose of 0, 0.6, 3.0, or 15 µmol/h for 5 h (A&C) or at the dose of 3.0 µmol/h for 0, 2, 5, or 8 h (B&D). Hypothalami were harvested and measured for the tissue contents of fumarate and succinate (A–D) and HIF2α protein (E&F). Bar graphs: * p<0.05; n = 6–8 per group. Error bars reflect mean ± SEM. (TIF) [file pbio.1001112.s008.tif]

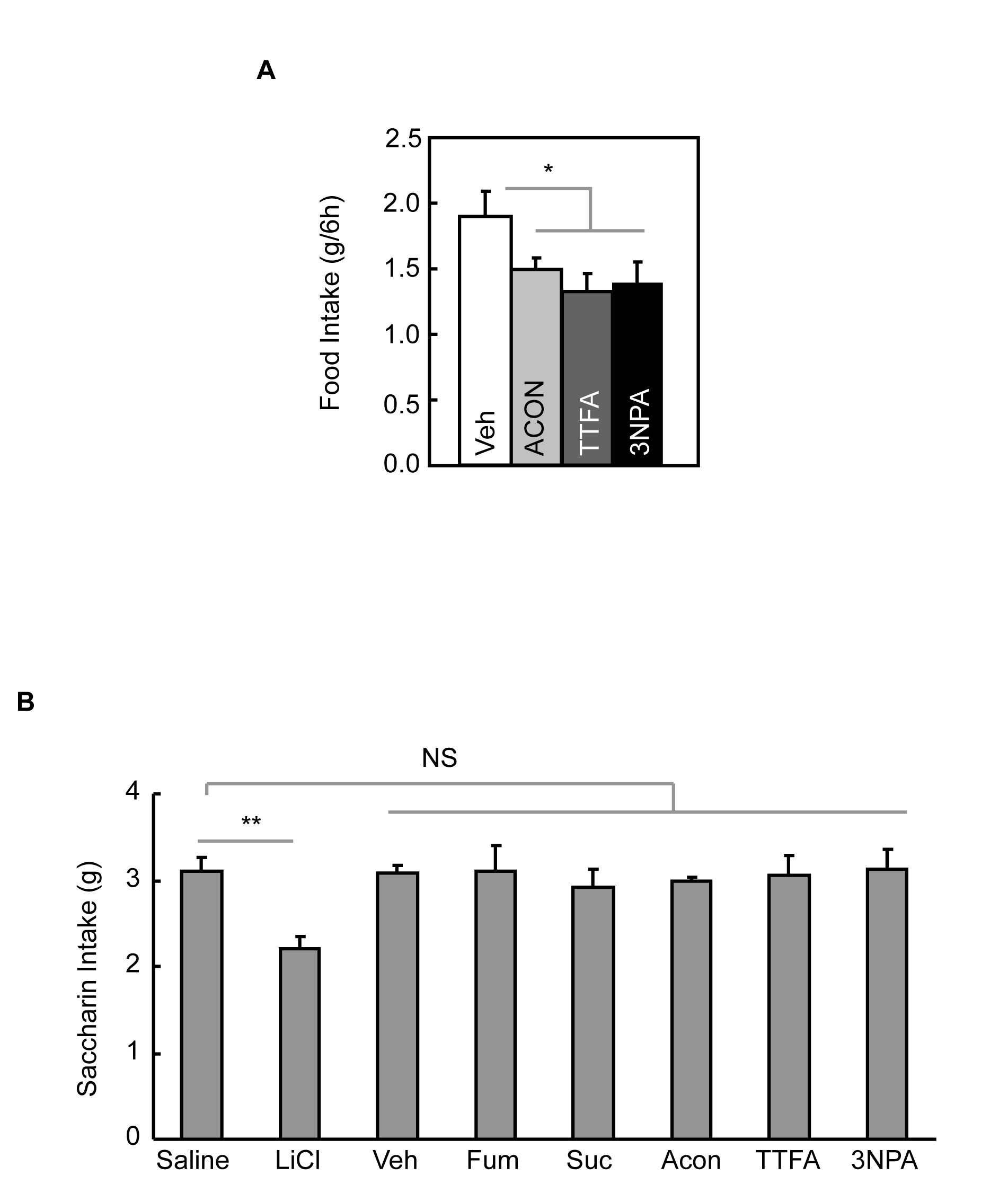

Supplement: Figure S9 — Food intake and taste aversion effects of pharmacologic chemicals. (A) Following 24-h fasting, C57BL/6 mice received third-ventricle injection of trans-aconitate (ACON), thenoyltrifluoroacetone (TTFA), 3-nitropropionic acid (3NPA), or vehicle (Veh). Food was provided to mice and food intake of mice was recorded. * p<0.05; n = 7–10 per group. Error bars reflect mean ± SEM. (B) C57BL/6 mice were habituated to experimental protocol for several days and then presented to 0.2% saccharine for 60 min after removing the drinking water for 23 h. After habitation, mice were ICV injected with an indicated drug and the vehicle (Veh) via pre-implanted third ventricle cannula, and subsequently had access to 0.2% saccharine for 60 min (Test 1). Intraperitoneal (IP) injection of LiCl and saline was used as a positive and negative control, respectively. After 4 d, mice were presented with 0.2% saccharine, and saccharine intake of mice during 60 min was measured (Test 2). Data represent 60-min saccharine intake in Test 2. ** p<0.01, ns, non-significant; n = 6–7 per group. Error bars reflect mean ± SEM. ACON, trans-aconitate; TTFA, thenoyltrifluoroacetone; 3NPA, 3-nitropropionic acid. (TIF) [file pbio.1001112.s009.tif]

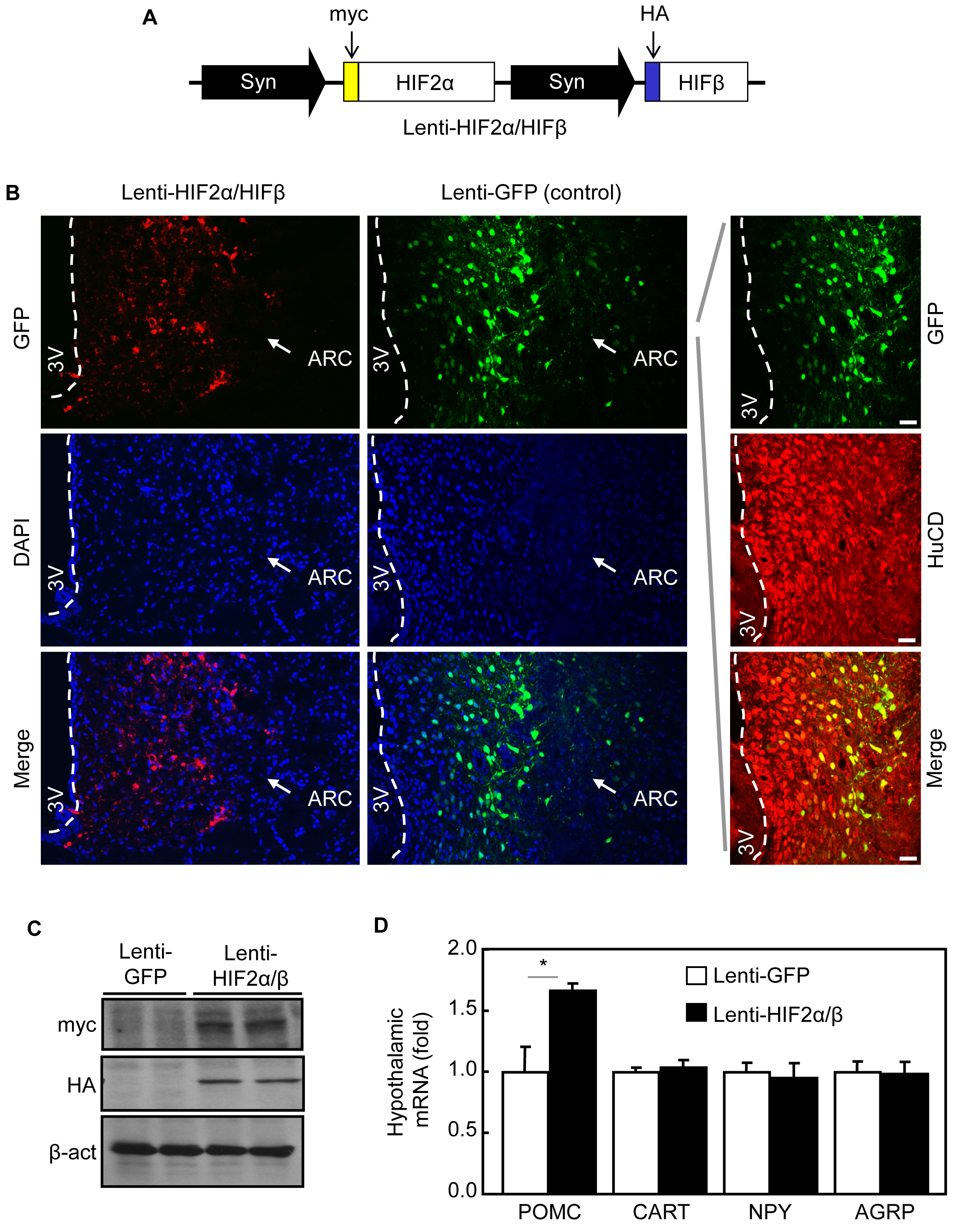

Supplement: Figure S10 — Lentivirus-directed hypothalamic HIF delivery up-regulates POMC gene. (A) Schematic map of lentivirus that co-expressed myc-conjugated HIFα (either HIF2α or HIF1α) and HA-conjugated HIFβ (Lenti-HIF2α/HIFβ) under the control of neuron-specific synapsin (Syn) promoter. Matched GFP-expressing lentiviral vector (Lenti-GFP) was used as a control. (B&C) C57BL/6 mice received intra-mediobasal hypothalamic injection of Lenti-HIFα/HIFβ or control Lenti-GFP. (B) Site-specific gene delivery was verified by GFP (green) and HA staining (red). DAPI nuclear staining (blue) reveals all cells in the sections. Right panels: GFP (green) and neuronal marker HuCD staining (red) are merged to indicate neuron-specific gene delivery (yellow). (C) Site-specific gene delivery was verified by Western blot analysis of myc and HA expression. Data shown in (B&C) were obtained from the mice injected with Lenti-HIF2α/HIFβ versus Lenti-GFP, but also represented similar patterns in mice injected with Lenti-HIF2α/HIFβ versus Lenti-GFP. ARC, arcuate nucleus; 3V, third ventricle; β-act, β-actin. Bar = 50 µm. (D) Chow-fed regular C57BL/6 mice that received bilateral MBH injections of Lenti-HIF2α/HIFβ or Lenti-GFP. At 2 wk post-injection, hypothalami were harvested for the measurement of POMC mRNA levels. * p<0.05; n = 5 per group. Error bars reflect mean ± SEM. (TIF) [file pbio.1001112.s010.tif]

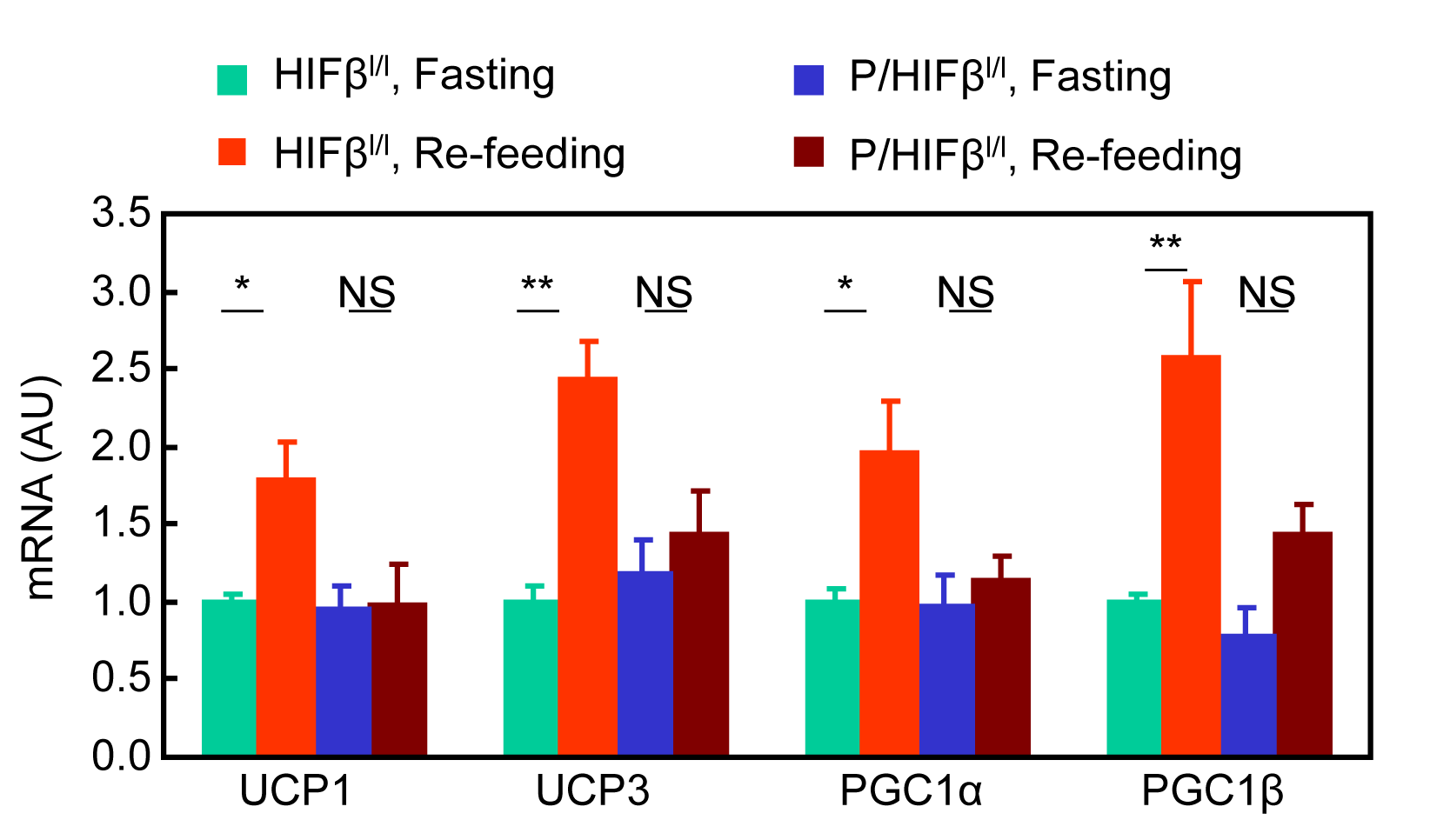

Supplement: Figure S11 — Thermogenic activities in POMC/HIFβlox/lox mice. Following 24-h fasting, POMC/HIFβlox/lox mice (P/HIFβl/l) and control HIFβlox/lox mice (HIFβl/l) received 6-h re-feeding versus continued 6-h fasting. Hypothalami were collected and analyzed for mRNA levels of indicated genes. * p<0.05, ** p<0.01, ns, non-significant; n = 5–8 per group. Error bars reflect mean ± SEM. (TIF) [file pbio.1001112.s011.tif]

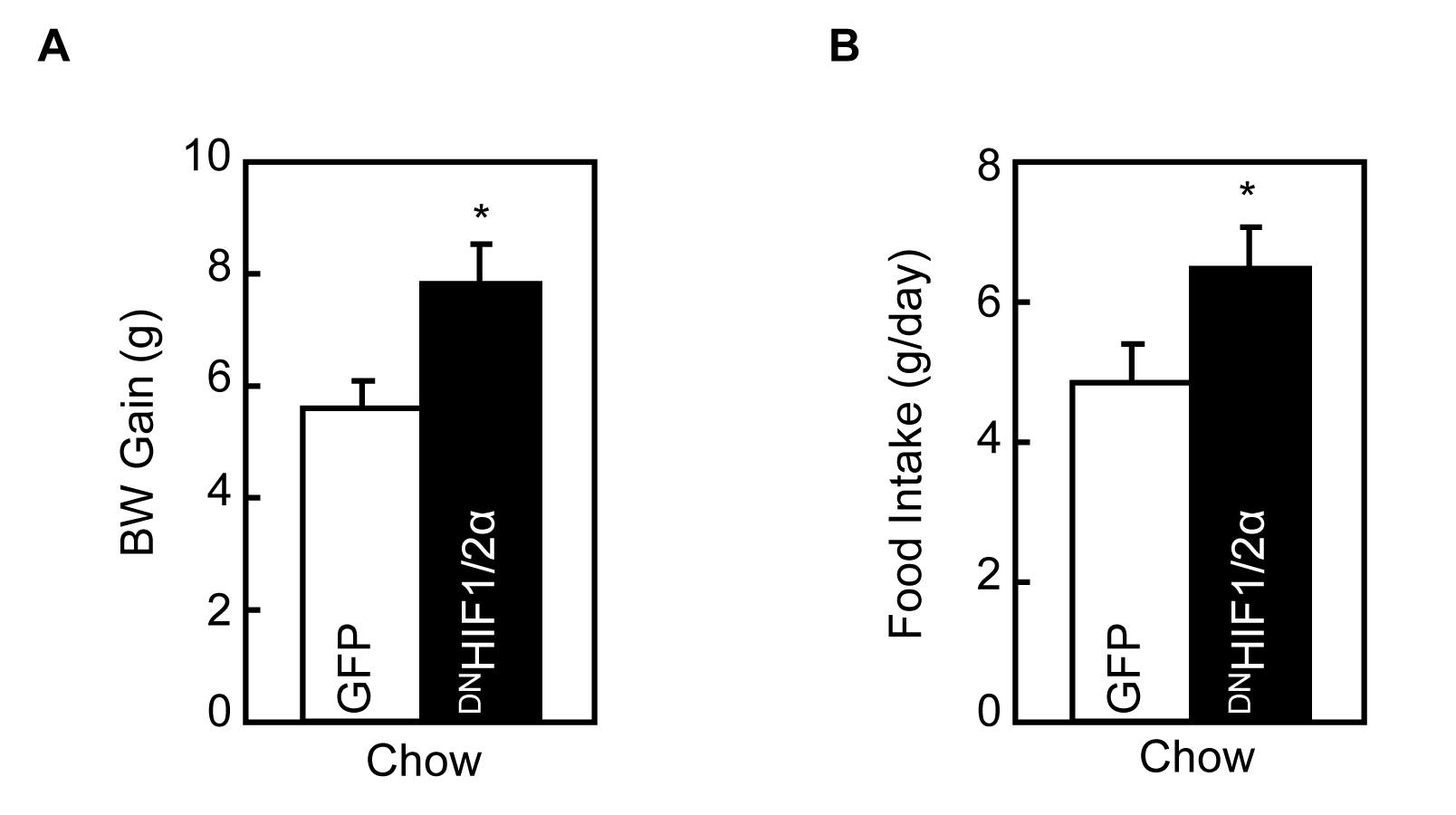

Supplement: Figure S12 — Effects of HIF on the metabolic phenotype in ob/ob mice. (A&B) Body weight-matched ob/ob mice (8 wk old) received intra-mediobasal hypothalamic injection of neuron-specific lentiviruses expressing dominant-negative HIF1/2α (DNHIF1/2α) or control GFP. Mice were monitored for body weight (BW) gain during 2-wk follow-up (A) and daily food intake during this follow-up period (B). * p<0.05; n = 5–6 per group. Error bars reflect mean ± SEM. (TIF) [file pbio.1001112.s012.tif]
