# Supplementary figures and images for: Correction: Hypoxia-Inducible Factor Directs POMC Gene to Mediate Hypothalamic Glucose Sensing and Energy Balance Regulation
Source: PLoS Biol. 2016 Mar 25;14(3):e1002428. doi: 10.1371/journal.pbio.1002428 (PMC4807785; doi:10.1371/journal.pbio.1002428)

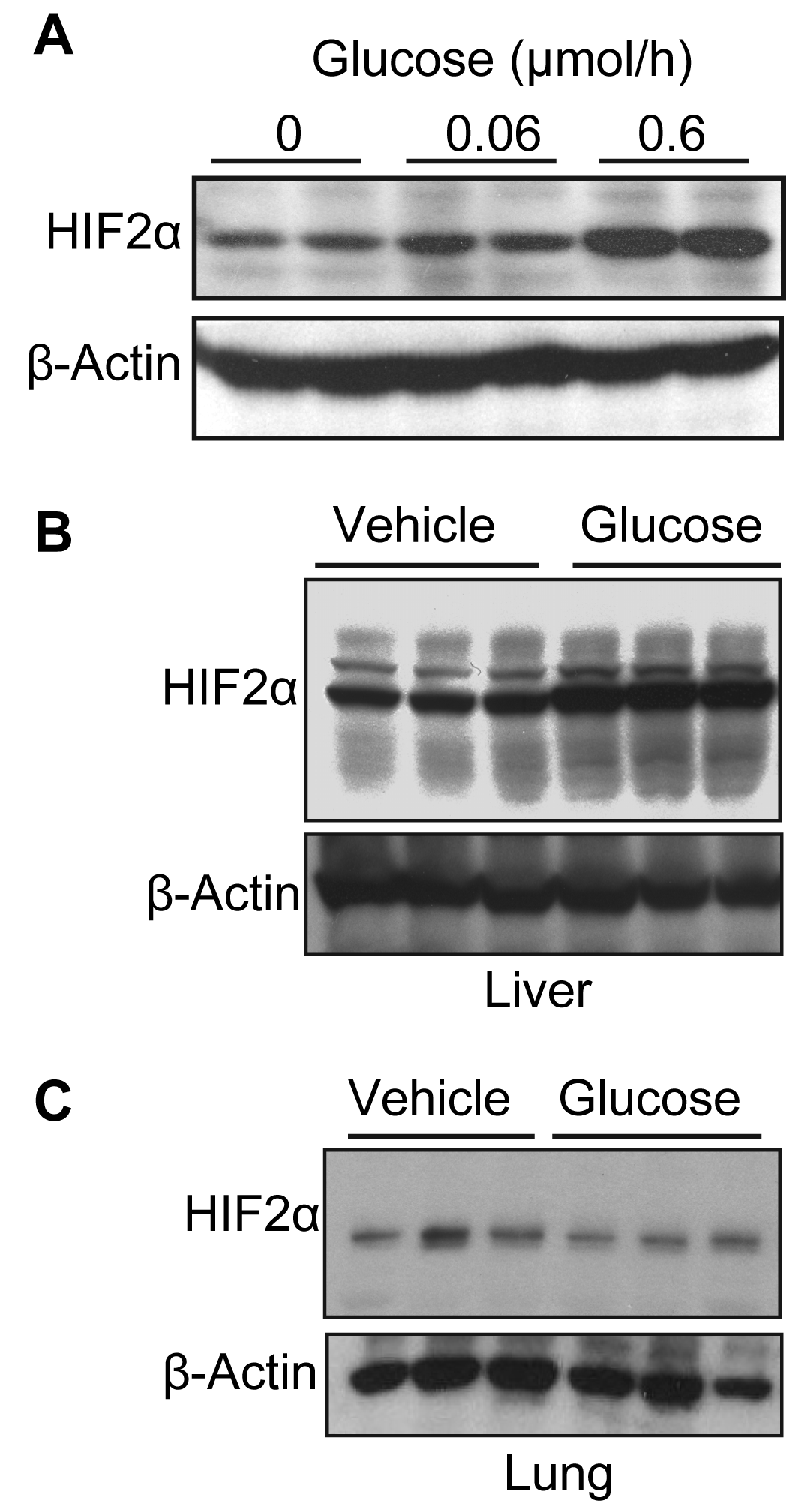

Supplement: S6 Fig — (A) Following 24-h fasting, C57BL/6 mice received third-ventricle injection of glucose at the indicated doses. HIF2α protein levels in the hypothalamus were examined by Western blots. β-actin was used as an internal control. (B&C) Following 24-h fasting, C57BL/6 mice received intraperitoneal injection of glucose (Glu) (2 g/kg body weight) or vehicle. HIF2α protein levels in the liver (B) and lung (C) tissues were examined by Western blots. β-actin was used as an internal control. (TIF) [file pbio.1002428.s001.tif]
